# The occurrence of cross-host species soil-transmitted helminth infections in humans and domestic/livestock animals: A systematic review

**DOI:** 10.1371/journal.pgph.0004614

**Published:** 2025-08-12

**Authors:** Uniqueky Gratis Mawrie, Riviarynthia Kharkongor, María Martínez Valladares, Stella Kepha, Sitara S. R. Ajjampur, Rajiv Sarkar, Rachel Pullan

**Affiliations:** 1 Department of Disease Control, Faculty of Infectious and Tropical Diseases, London School of Hygiene and Tropical Medicine, London, United Kingdom; 2 Indian Institute of Public Health Shillong, Shillong, Meghalaya, India; 3 Departamento de Sanidad Animal, Instituto de Ganadería de Montaña (CSIC-Universidad de León), León, Spain; 4 Eastern and Southern Africa Centre of International Parasite Control, Kenya Medical Research Institute, Nairobi, Kenya; 5 The Wellcome Trust Research Laboratory, Division of Gastrointestinal Sciences, Christian Medical College, Vellore, Tamil Nadu, India; University of Oslo Faculty of Medicine: Universitetet i Oslo Det medisinske fakultet, NORWAY

## Abstract

Zoonotic soil-transmitted helminths (STH), including *Ancylostoma ceylanicum*, *Ancylostoma caninum*, *Ancylostoma braziliense*, *Trichuris vulpis*, *Trichuris suis*, and *Ascaris suum*, are increasingly recognised as potential sources of human infection. Additionally, animals can act as carriers or reservoirs for human STH species. However, the extent of cross-host infection remains poorly understood, primarily due to reliance on morphological diagnostics. This review compiles data on the occurrence of cross-host STH infections, highlighting zoonotic STH in humans and human STH species in domestic and livestock animals. Following PRISMA guidelines, PubMed, Medline, and Web of Science were systematically searched without restriction on publication date, covering records available from inception to December 2024, with the earliest retrieved study published in 1942. Inclusion criteria encompassed studies on cross-host STH infections confirmed by molecular methods. Exclusion criteria included experimental infection studies, studies involving wildlife, and those that did not find cross-host infection. Two independent reviewers assessed bias using Appraisal tool for Cross-sectional studies (AXIS) and Joanna Briggs Institute appraisal tools. The protocol is registered with PROSPERO (CRD42024519067). The review screened 4197 titles and abstracts and included 51 studies. *Ancylostoma ceylanicum* was the commonest zoonotic STH reported, predominantly in Southeast Asia. Human STH species (*Ancylostoma duodenale, Necator americanus, Trichuris trichiura* and *Ascaris lumbricoides*) were found in dogs, cats, and pigs. Studies examining both humans and animals together in shared environments showed STH presence in both populations. Case studies revealed gastrointestinal and dermatological effects in humans particularly infected with zoonotic hookworms. This systematic review highlights STH cross-host species infections underscoring the need for further One health epidemiological investigations of humans and domestic/livestock animals in sympatric environments to better understand the burden and explore the transmission dynamics of cross-host STH infections.

## Background

The soil-transmitted helminths (STH) in humans are neglected tropical diseases caused commonly by *Ascaris lumbricoides*, *Trichuris trichiura* and hookworms (*Ancylostoma duodenale, Necator americanus*). The World Health Organisation has specified aims to eliminate STH as a public health problem in 96% of endemic countries by 2030, targeting a reduction in heavy-to-moderate intensity infection prevalence in children to below 2% by preventive chemotherapy [1]. While widespread deworming initiatives have successfully reduced morbidity in most settings [2], persistent environmental contamination may contribute to continued reinfection, raising questions around sustainability [3,4]. In order to address environmental contamination, focus has been primarily directed towards improvements in water, sanitation, and hygiene (WASH) infrastructure and behaviour change communication to prevent human faecal contamination [5]. However, evidence suggest WASH’s effectiveness is low-to-moderate [6–8] and it may not effectively address all sources of environmental contamination.

Zoonotic helminths are globally prevalent in animals, but the frequency of their occurrence in humans and its public health implications remain largely unexplored. Most notably, dogs and cats serve as hosts for the hookworm species *Ancylostoma ceylanicum*, *Ancylostoma caninum*, *Ancylostoma braziliense*, in addition to *Trichuris vulpis*, while pigs host *Ascaris suum* and *Trichuris suis* - all of whom have zoonotic potential. Limited evidence of their presence in humans is due to the inability of morphological egg identification (until recently, the primary diagnostic tool) to differentiate between species. Nevertheless, genetic analyses suggests potential cross-host species infection of *Ascaris* and *Trichuris* between humans and pigs [9–12], including hybridisation between *A. suum* and *A. lumbricoides* [13,14]. Furthermore, it is also possible that animals can act either as carriers/transport hosts [15,16] or reservoirs of human STH species, potentially influencing the transmission dynamics.

Given that dogs’ and cats’ faeces commonly contaminate soil in areas where they roam freely [17–20], and that pigs are reared closely with humans in many low-and-middle-income settings, it is important to ascertain their role in maintaining STH transmission in human populations. Considering the potential challenges to disease control, posed by animal reservoirs, this study systematically reviews evidence of cross-host species infections of zoonotic STH (*A. ceylanicum*, *A. caninum*, *A. braziliense*, *T. vulpis*, *A. suum*, and *T. suis*) in humans and human STH (*A. lumbricoides, T. trichiura*, *A. duodenale* and *N. americanus*) in domestic/livestock animals. It aims to understand the geographical distribution and the extent of cross-host species infections to explore the role of animals in the transmission of STH in endemic settings.

## Methods

### Search strategy and selection criteria

Following the Preferred Reporting Items for Systematic Review and Meta-Analyses (PRISMA) guidelines [21] (S1 Checklist), a systematic review on cross-host species infections of STH between humans and animals (domestic/livestock) was conducted. The protocol of this systematic review was registered with PROSPERO (registration ID CRD42024519067). The review was carried out by two independent reviewers, UM and RK. UM formulated the research questions, developed inclusion and exclusion criteria, and build the search strategy. Titles/abstracts and full texts were screened independently by both reviewers, with discrepancies resolved through discussion. Risk of bias assessments were also conducted independently. The initial search was conducted between June and August 2023, with an updated search conducted in December 2024 (Detailed timelines are described in S1 Table). UM took the lead in the interpretation and reporting the findings.

### Database search

Three databases-PubMed, Medline, and Web of Science, were searched without applying date restrictions for studies published since inception to December 2024, with the earliest retrieved study published in 1942. The search strategy was designed to include evidence on “Cross-host species infection” between humans and animals defined as the occurrence of zoonotic STH species, namely *A. ceylanicum*, *A. caninum*, *A. braziliense*, *A. suum*, *T. vulpis* and *T. suis* in humans. It also included occurrences of human STH species that is, *A. lumbricoides*, *T. trichiura, A. duodenale* or *N. americanus*, in domestic/livestock animals. BOOLEAN operators ‘OR’ and ‘AND’ were used to refine the search strategy. The list of search terms and details of the search strategy applied to extract studies from each database is described in S1 Table.

### Study selection

Original studies employing molecular methods to confirm STH species in cross-host species infections were considered for inclusion. Only studies using molecular diagnostic methods were included in this review due to the limitations of conventional microscopy, such as the Kato-Katz method, which cannot distinguish between morphologically similar STH species. For example, overlapping egg sizes and morphological similarities, between *A. lumbricoides* and *A. suum* [22], or *T. trichiura* and *T. vulpis* [23] can result in misidentification. Since this review aims to establish the occurrence (not necessarily the prevalence) of cross-host species infections, both community-based studies and studies conducted in hospital settings were included. We included only papers that explicitly reported cross-host species infections, excluding those that investigated but did not find evidence of such infections. Experimental studies (artificially-induced infections), studies reporting helminths in wildlife animals, studies that did not use molecular methods were excluded. Other forms of publications such as editorials, or review papers were also excluded but bibliography was checked for further references. Unpublished and grey literature were not assessed. *Strongyloides stercoralis*, although classified as an STH, was excluded from this review due to its unique features: under-reporting due to auto-infection [24], diagnostic limitations related to Kato-katz method [25], and Ivermectin as the preferred treatment and preventive deworming [26]; hence was excluded from this review.

### Data extraction

The studies identified from all the three databases were exported to Zotero (Zotero 6.0.36) [27] for management and referencing. Rayyan software [28] was used for study organisation and duplicate detection, with manual screening. After removing duplicates, titles and abstracts were screened. Studies with unclear eligibility were included for further assessment. Full-texts of eligible studies were retrieved.

Information on the year of publication, species detected, animal hosts, country, diagnostic method used, sample size, number of samples tested by morphological examination, number of samples tested by molecular method, gene targets, number of samples tested as positive, prevalence (with 95% confidence intervals, when applicable), clinical symptoms and case description (for case studies) were extracted (S1 Data). If participants had travelled to an endemic region, then the place of origin and the place travelled were also recorded. No pooled analysis was conducted.

Data were extracted and synthesised as presented in the original studies. When percentages were not reported but sufficient raw data were available, corresponding percentages were calculated. Conversely, when only percentages were provided and the denominators were clearly stated, the absolute number of cases was derived. Instances of mixed infections were recorded as reported; when specific species were not identified, these were noted accordingly. For case reports lacking details such as travel history or clinical symptoms, missing information was acknowledged.

### Quality assessment

Risk of bias was assessed using the Appraisal tool for Cross-sectional studies (AXIS) [29] for community-based studies and the Joanna Briggs Institute (JBI) Critical Appraisal tools for cross-sectional studies and case reports [30] for hospital-based studies. AXIS consists of 20 items evaluating study design, reporting quality, and bias risk. The JBI tool for cross-sectional studies assesses sample criteria, subject descriptions, measurement validity, confounding factors identification, and statistical analysis appropriateness. For case reports, the JBI tool evaluates clarity of case descriptions, suitability of diagnostic methods, consideration of confounding factors, and reliability of conclusions. All eligible studies were included regardless of the quality score.

## Results

### Search results

**Fig 1:**
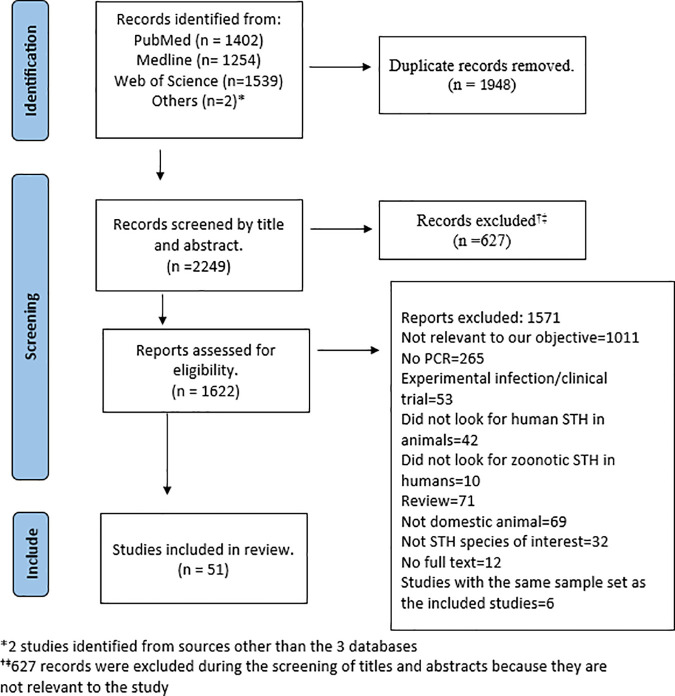
Flow diagram showing the screening and selection of studies using the Preferred Reporting Items for Systematic Reviews and Meta-Analyses (PRISMA).

The process of selecting eligible studies using the PRISMA guidelines is presented in Fig 1.

The search identified 4197 studies. The distribution of records retrieved from each database is detailed in S2 Data. After removing 1948 duplicates, 2249 records were screened for titles and abstracts, of which 627 were excluded. A total of 1622 full-text articles were evaluated for eligibility against the pre-determined eligibility criteria. Fifty-one studies were included in this review, after excluding 1011 studies that were not relevant to the study objective, 265 studies that did not use molecular methods for identification of STH species, 53 experimental infection studies, 32 studies that only included unrelated helminth species, 69 studies involving only wild animals and 12 records for which full-text was not available (Fig 1). The reason for excluding the papers after full-text assessment is explained in the S3 Data. The 12 records for which the full texts were unavailable were published between 1949 and 2001. Given the age of these publications and the likelihood that they did not employ molecular diagnostic methods for species confirmation, efforts to contact the authors were not undertaken.

### Study characteristics

**Fig 2:**
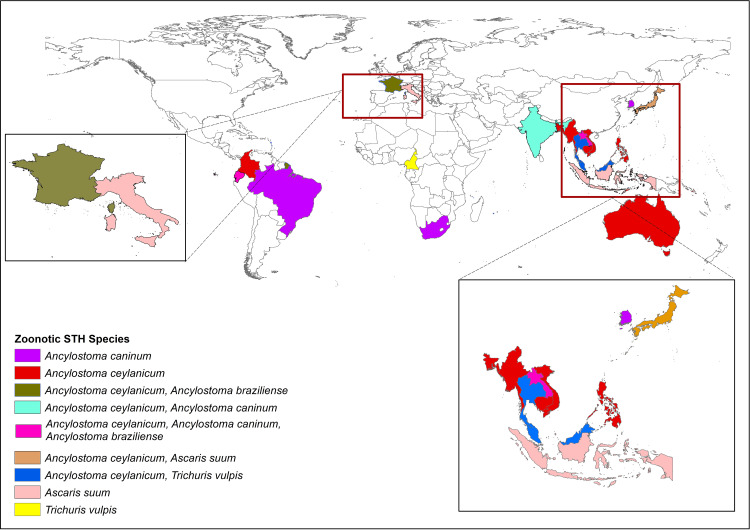
Distribution of studies reporting the occurrence of zoonotic STH across Southeast Asia, South Asia, Oceania, Africa, Europe and South America. The country borders shapefile used as the base layer is the wb_countries_admin0_10m dataset, available from https://datacatalog.worldbank.org/search/dataset/0038272, and is licensed under the Creative Commons Attribution 4.0 International (CC BY 4.0) license.

**Fig 3:**
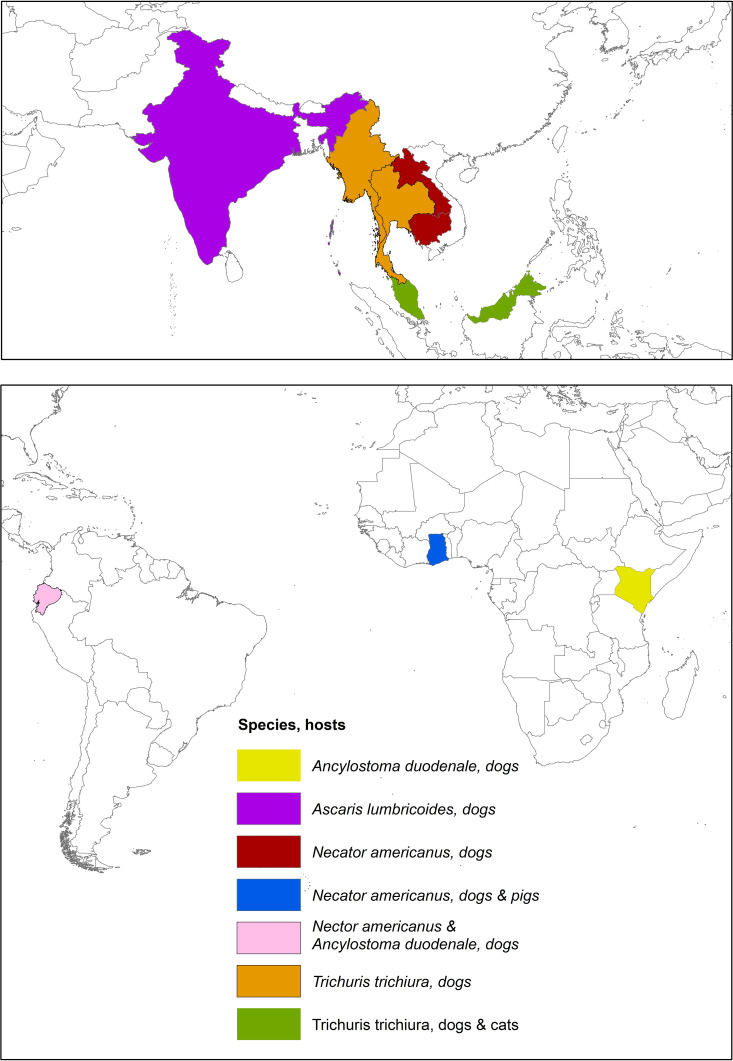
Distribution of studies reporting the occurrence of human STH species in animal hosts. The country borders shapefile used as the base layer is the wb_countries_admin0_10m dataset, available from https://datacatalog.worldbank.org/search/dataset/0038272, and is licensed under the Creative Commons Attribution 4.0 International (CC BY 4.0) license.

Thirty-five (68.6%) of the 51 studies that met the eligibility criteria were community-based while 16 (31.4%) studies were conducted in hospital settings. Majority of the studies (96·1%, 49 of 51) were published in the last decade (2010–2022) (S1 Fig). Studies conducted across 28 countries revealed the presence of cross-host species STH infections in humans and animals. Predominantly, 62·7% (32 of 51) of these studies were from 12 countries in Southeast Asia (SEA), with a smaller proportion from South America (11·8%, 6 of 51), Europe (9.8%, 5 of 51), Africa (7.8%, 4 of 51), South Asia (7.8%, 4 of 51) and Oceania (3·9%, 2 of 51) (Figs 2, 3, S2 and S3).

Among the community-based studies, the majority (74.3%, 26 of 35) utilised cross-sectional designs. A smaller percentage were integrated into larger programs (5·7%, 2 of 35) [31,32], while others employed alternative designs, including cluster-randomised trial (2.8%, 1 of 35) [33], efficacy trial of treatment or drugs (5.7%, 2 of 35) [34,35], testing/validation of diagnostic tools or treatment (5.7%, 2 of 35) [36,37], cohort study design (2.8%, 1 of 35) [38], or retrospective analysis of archived samples from previous studies (2.8%, 1 of 35) [39]. Rural populations were the focus of the majority of community-based studies (65.7%, 23 of 35) [23,40–47], with some studies (11.4%, 4 of 35) specifically targeting tribal or indigenous communities [40,41,43,44]. Additionally, 22·8% (8 of 35) focused on children, particularly preschool and school-aged groups, with a subset conducted in daycare centres and schools (5·7%, 2 of 35) [36,48,49]. Other specific study locations included communities around temples [50], refugee camps [31,32], and tea-growing communities [51]. Site selection methods varied, including accessibility-based approaches [41,43,45,52] and consideration of contact with domestic animals [45,51]. Sampling methodologies encompassed simple random sampling of households [35,52], random selection of villages/schools [43,53], random sampling of participants [54], purposive [47] and convenience sampling [55] (Table 1)

**Table 1:** Characteristics of eligible community-based studies reporting zoonotic STH species in humans and/or human-associated STH species in animals.

Citation	Country	Study design/ participants	Type of study	Selection of isolates for molecular testing	Number tested	PCR* method	Gene targets	Species in humans	Species in animals	Quality score ^§^
George et al (2016) [34]	Brazil, Cambodia, Cameroon, Ethiopia, Tanzania, Vietnam	Treatment efficacy trials, children STH egg positive by McMaster	Drug/treatment efficacy trial	Random sample (20–40 samples per site)	207 in total across 6 countries	Semi-nested PCR, PCR-RFLP^&^	ITS^-1, 2 and 5·8s region	- ***T. vulpis***: 3·4% (7/207)^¶^ - ***A. suum***: nil - *A. lumbricoides*: 34·2% (71/207) - *T. trichiura*: 42·0% (87/207) - *N. americanus*: 35·2% (73/207) - *A. duodenale*: 19·3% (40/207) -Mixed infections(*N. americanus* and *A. duodenale*)-4.3% (9/207)	*Not assessed*	18
Chang et a^£^(2020) [56]	Cambodia	Worm expulsion study in one village with high hookworm prevalence	Cross-sectional	All adult hookworms recovered from 9 people	65 adult worms	PCR	COX1^±^	- **A. *ceylanicum***: 9·3% (6/65) *- N. americanus*:90·7% (59/65) -Mixed infections (*N. americanus* and *A. ceylanicum*): 3 patients	*Not assessed*	18
Colella et al (2021) [35]	Cambodia	Treatment efficacy trial, individuals aged over 6 years from ten villages positive by standard faecal flotation	Drug/treatment efficacy trial	All samples collected	151 human faecal samples	Multiplex qPCR^#^	ITS-1, 2 region	- ***A. ceylanicum***: 5·3% (8/151) *- N. americanus*: 86·8% (131/151) - Mixed infection- 7·9% (12/151)	*Not assessed*	18
Inpankaew et al (2014) [42]	Cambodia	Cross-sectional study of 67 households randomly selected, 218 and 94 dogs individuals enrolled, egg positive by microscopy	Cross-sectional	All samples collected	218 human faecal samples, 94 dog faecal samples	PCR, PCR-RFLP	ITS-1, 2 and 5·8s region	*Hookworm spp:* 56·9% (124/218) *-* ***A. ceylanicum:*** 46·0% (57/124) *- N. americanus:* 47·6% (59/124) *-A. duodenale:* 0·8% (1/124) *-* Mixed infection *(N. americanus* and *A. ceylanicum):* 3·2% (4/124) *-* Mixed infection (*A. ceylanicum* and *A. duodenale)-* 1·6% (2/124) *-* Mixed infection (*N. americanus*, *A.ceylanicum* and *A. duodenale):* 0·8% (1/124)	*- Hookworm spp:* 95.7% (90/94) - *A. ceylanicum*: 90·0% (81/90) - *A. caninum*: 5·6% (5/90) - *A. ceylanicum* and *A.caninum*: 3·3% (3/90) - A*. ceylanicum* and ***N. americanus***: 1·1% (1/90)	18
Sears et al (2022) [55]	Ecuador	Cross-sectional study samples from a convenience subsample of preschool, schoolchildren and daycare staffs from two provinces	Cross-sectional	Single samples from 230 individuals	230 human faecal samples	Multi parallel qPCR	ITS-1, 2 and 5·8s region	*-* ***A. ceylanicum***: 2·6% (6/230) *- N. americanus:* 37·0% (85/230)	*Not assessed*	18
Calvopina et al (2024) [40]	Ecuador	Study conducted in a village inhabited by indigenous population. Single faecal samples collected from humans and dogs. Presence of eggs or larvae was assessed using formalin-ether concentration	Cross-sectional	All samples collected	54 human faecal samples, 79 dog faecal samples	Multi-parallel qPCR, conventional PCR	ITS-1	***- A. ceylanicum***: 14.8% (8/54) ***- A. caninum***: 11.1% (6/54) ***- A. braziliense***: 1.9% (1/54) *- A. duodenale*: 31.5% (17/54), *- N. americanus*: 14.8% (8/54) - Mixed infection (anthroponotic/zoonotic species): 11.0% (6/54)	***- N. americanus***: 12.4% (10/79) ***- A. duodenale***: 6.3% (5/79) *- A. ceylanicum*: 78.5% (62/79) *- A. caninum*: 49.4% (39/79) *- A. braziliense*: 21.5% (17/79) - Mixed infections (anthroponotic/zoonotic species): 13.9% (11/79)	15
Aguilar-Rodríguez et al (2024) [39]	Ecuador	Retrospective analysis of archived samples from previous studies.	Retrospective analysis of archived samples	Microscopy positive samples from 4 studies and microscopy negative samples from 5 studies	132 faecal samples. 69 samples which were microscopy positive and 63 samples which were microscopy negative	Multi-parallel qPCR	COX1	***- A. ceylanicum****: 19.7%* (26/132) *- A. duodenale: 34.9%* (46/132) *- N. americanus: 18.2%* (24/132) *- S. stercoralis:* *6.8%* (9/132) *- T. trichiura: 15.9%* (21/132) *- A. lumbricoides: 71.2%* (94/132) - Mixed infection (with any STH species): 81.8% (108/132)	*Not assessed*	17
Boyko et al (2020) [16]	Ghana	Faecal samples collected from dogs and pigs, hookworm eggs positive by Kato–Katz method	Cross-sectional	Microscopy positive faecal samples collected from 64 dogs and 20 pigs	43 dog and 9 pig hookworm positive faecal samples	PCR	ITS-2, COX1	*Not assessed*	- ***N. americanus***: *D*ogs: 47·0% (20/43) Pigs: 56·0% (5/9)	18
Traub et al (2002) [51]	India	Survey in tea-growing communities, including adults and children (staff and labour populations, excluding executives), eggs assessed by sedimentation and centrifugal flotation	Cross-sectional	Dogs-*Ascaris* eggs which are microscopy positive. Human- *Ascaris* eggs, adult A. lumbricoides worm as positive controls	5 human faecal samples, 31 dog faecal samples	PCR-RFLP	ITS-1, 2 and 5·8s region	*Not assessed*	All 31 dog-derived *Ascaris* egg samples exhibited a digestion pattern that matched those described for human-derived ***Ascaris*.** 5 dog-derived *Ascaris* eggs exhibited 100% homology with those found in *Ascaris* eggs from humans and the adult worm from Assam -Mixed infections (with >1 zoonotic species): 99.0%	18
George et al (2015) [44]	India	Survey of children 1–15 years, eggs positive by saline wet microscopy method	Cross-sectional	A subset of 50 randomly selected microscopy positive samples	50 human faecal samples	Semi-nested PCR, PCR-RFLP	ITS-1, 2 and 5·8s region	*- Hookworm spp:* 82·0% (41/50) *-* ***A. ceylanicum****: 5·0% (2/41)* *- N. americanus: 95·0% (39/41)* *- A. duodenale: 15·0% (6/41)* -Mixed infections (*A. duodenale* and *N. americanus*): 14.6% (6/41)	*Not assessed*	18
George et al (2016) [33]	India	Part of a community-based cluster randomised control trial, faecal samples of humans, dogs and soil samples from 9 clusters, egg positive by saline wet mount	Cluster randomised trial	146 of 711 positive individuals that were positive by microscopy 77 of 90 dogs that were positive by microscopy	143 human faecal samples, 77 dog faecal samples	PCR-RFLP	ITS-1,2 and 5·8s region	- *Hookworm spp*.: 83·2% (119/143) *-* ***A. caninum***: 16·8% (20/119) – *N. americanus:100·0% (119/119)* *- A. duodenale:* 8·4% (10/119) *-*Mixed infections (with any of the hookworms): detected, but numbers not specified	- Hookworm DNA: 88·3% (68/77) *- A. caninum:* 76·5% (52/68) *- A. ceylanicum:* 27·9% (19/68) *-* Mixed infections (*A. caninum* and *A. ceylanicum*): 4·4% (3/68)	*18*
Agustina et al (2023) [47]	Indonesia	Cross-sectional study, samples collected from pig farmers using purposive sampling. Flotation concentration method used for the detection of *Ascaris* spp. eggs	Cross-sectional	All samples collected	239 human faecal samples	PCR	COX1	***A. suum***: 1.25% (3/239)	*Not assessed*	15
Mulinge et al (2020) [57]	Kenya	Single canine faecal samples collected from the environment	Cross-sectional	78 of 490 faecal samples that were positive by microscopy were randomly selected	PCR products obtained from 70 of 78 dog faecal samples	PCR, PCR-RFLP	ITS-1,2, 5·8s and 28S region	*Not assessed*	- *A. caninum*: 84·3% (59/70) *- A. braziliense:* 14·3% (10/70) ***- A. duodenale:*** 1·4% (1/70)	*18*
Conlan et al (2012) [52]	Lao PDR	A survey conducted in one 6 randomly selected villages (one district selected randomly from each of the four provinces), with 14 households chosen per village. All household members aged 6 and above were invited to participate, egg-positive by formalin-ether concentration technique. Dog faecal samples collected from household with dogs.	Cross-sectional	Human-46 randomly selected from microscopy positive samples;17 of 46 (successfully amplified) Dogs-23 of 94 selected;18 of 23 successfully amplified.	46 human faecal samples, 23 dog faecal samples	PCR	ITS-1,2 and 5·8s region	*-* ***A. ceylanicum***: 17·6% (3/17) *- N. americanus:* 82·4% (14/17)	- ***N. americanus:*** 5·6% (1/18) *- A. ceylanicum:* 38·9% (7/18) - *A. caninum:* 11·1% (*2/18)* - *A. braziliense:* 5·6% (1/18) - Mixed infections (*A. ceylanicum* and *A. caninum*): 22.2% (4/18) -Mixed infections (*A. ceylanicum* and *N. americanus*): 16.7% (3/18)	*18*
Sato et al (2010) [58]	Lao PDR	Single faecal sample collected from each of individuals, aged 8–60 years, hookworm egg positive by Kato-Katz method	Cross-sectional	All samples recovered from 203 individuals	203 human faecal samples	PCR	ITS-1,2 and 5·8s region	*- N. americanus:* 5·9% (12/203) *- Ancylostoma spp.:* 9·4% (19/203) *-* Mixed infections (*N. americanus* and *Ancylostoma spp.*)*: 0·5% (1/203)* ***Sequencing-****9/20 samples, and the amplicon of the adult A. duodenale, were successfully sequenced* *-* ***A. caninum:*** *33·3% (3/9)* *-* ***A. ceylanicum:*** *11·1% (1/9)* *- A. duodenale: 55·6% (5/9)*	*Not assessed*	*18*
Ash et al (2017) [37]	Lao PDR	Participants from a village were invited to provide faecal samples. Village dog samples were collected opportunistically	Testing/validation of a diagnostic tool	Human- a subset of 31 hookworm samples positive by microscopy Dog- all 9 samples	31 human faecal samples, 9 dog faecal samples	PCR	ITS-1,2 and 5·8s region	- ***A.* ceylanicum**: 6·4% (2/31) *- N. americanus:* 70·9% (22/31) *- A. duodenale:* 6·4% (2/31) *-* ***A. braziliense***: 19·4% (6/31) *- N. americanus* and *A. ceylanicum):* 3·2% (1/31) - Mixed infections (*N. americanus* and *A. ceylanicum*): 3.2% (1/31)	*-* ***N. americanus***: 22·2% (2/9) *- A. ceylanicum:* 44·4% (4/9) *- A. caninum:* 44·4% (4/9) *- A. ceylanicum* and *A. caninum: 11·1% (1/9)* - Mixed infections (*A. ceylanicum* and *A. caninum*): 11.1% (1/9)	*18*
Niamnuy et al (2016) [54]	Lao PDR, Thailand	Cross-sectional study in 3 districts, dogs and cats from participating households were randomly selected.	Cross-sectional	5 samples were cultured for PCR	5 hookworm larvae	PCR	ITS-1,2 and 5·8s region	*-* ***A. ceylanicum***: 20·0% (1/5) *- N. americanus:* 80·0% (4/5)	*Not assessed*	*18*
Chin et al (2016) [41]	Malaysia	Cross-sectional study in two ethnic groups in 4 accessible villages	Cross-sectional	All 186 samples collected	186 human faecal samples	Two step semi-nested PCR	ITS-2, 28S region	*-* ***A. ceylanicum***: 4·3% (8/186) *- N. americanus:* 20·4% (38/186) *-* Mixed infections (*N. americanus* and *A. ceylanicum)*-1·1% (2/186)	*Not assessed*	*18*
Ngui et al (2012) [45]	Malaysia	Study in 8 remote villages selected based on high hookworm prevalence and accessibility	Cross-sectional	Human- 47 of 58 microscopy-positive samples were successfully amplified Dogs and cats- 50 of 65 microscopy-positive samples were successfully amplified.	47 human faecal samples, 50 dog and cat faecal samples	PCR, Two-step semi-nested PCR	ITS-2, 5·8s and 28S region	*-* ***A. ceylanicum***: 12·8% (6/47) *- N. americanus:* 76·6% (36/ 47) - Mixed infections (*A. ceylanicum* and *N. americanus)*: 10·6% (5/47)	*- A. caninum (dogs):* 52·0% (26/50) *- A. ceylanicum* (cats & dogs): 46·0% (23/50) *- A. braziliense* (cats): 2·0% (1/50)	*17*
Mohd-Shaharuddin et al (2019) [43]	Malaysia	A cross-sectional study in indigenous communities, 5 accessible villages selected by convenience sampling, individuals were invited to voluntarily participate	Cross-sectional	Human, dog and cat-Samples positive by microscopy	240 human faecal samples, 74 dog and cat faecal samples	Two step semi-nested PCR	SSUrRNA^||^	*-* ***T. vulpis***: 1·3% (3/240) *- T. trichiura*: 98·7% (237/240)	*-* ***T. trichiura***: 56·8% (42/74) - *T. vulpis:* 43·2% (32/74) Microscopy -ve samples confirmed to be -ve by PCR **Sequencing:** 99%-100% homologous to ***T. trichiura*** and *T. vulpis (*NCBI database)	*18*
Dunn et al (2020) [59]	Myanmar	A cross-sectional study among residents of delta region of Myanmar, single stool sample of participants were tested egg positive by Kato-Katz method	Cross-sectional	All 648 samples collected from participants	648 human faecal samples	qPCR	ITS region	*-* ***A. ceylanicum****: 4·6%* [(95% CI-3·15-6·54) (30/648)] *- A. lumbricoides:* 8·8% [(95% CI 6·73–11·25) (57/648)] *- T. trichiura:* 22·8% [(95% CI 19·66–26·27) (148/648)] *- N. americanus:* 22·7% [(95% CI 19·51–26·11) (147/648)] *- A. duodenale:* 0·2% [(95% CI 0·00–0·86) *(1/648)]* - Mixed infections (two-three species): 28.8% (84/292)	*Not assessed*	*18*
Htun et al (2021) [60]	Myanmar	A cross-sectional study of dogs in 11 locations, stool samples assessed by sedimentation and flotation and McMaster methods	Cross-sectional	Only samples that were morphologically positive for hookworm and whipworm eggs	166 *Ancylostoma spp.* positive, 15 *Trichuris spp*. positive samples	PCR	COX1, SSU rRNA	*Not assessed*	*-* ***T. trichiura***: 26·7% (4/15) *- T. vulpis:* 86·7% (13/15) - Mixed infections (*T. trichiura* and *T. vulpis*): 13·3% (2/15) *- A. ceylanicum:* 72·2% (120/166)	*18*
Aung et al (2017) [46]	Myanmar	Faecal samples from individuals 5–60 years in three rural areas, hookworm egg positive by ethyl acetate concentration technique	Cross-sectional	Only samples that were positive for hookworm eggs	21 human *Hookworm spp.* positive samples	PCR	ITS-1,2 and 5·8s region	Sequence results- 8 and 3 samples showed 99–100% similarity to *N. americanus* with ***A. ceylanicum*** respectively	*Not assessed*	*18*
O’Connell et al (2018) [31]	Myanmar, Thailand	Part of a larger program, individuals >6 months in refugee camps	Intergrated to a larger program	All samples included at the baseline of the study	1548 human faecal samples	Multiparallel qPCR, PCR-RFLP.	ITS-1,2 and 5·8s region	-***A. ceylanicum***: 5·4% (83/1548) *-N. americanus*-25·4% (393/1548)	*Not assessed*	*18*
Aula et al (2020) [61]	Philippines	Stool samples from humans were collected across 18 locations in a previous study. Faeces from dogs were collected in a separate survey, egg positive by Kato-Katz method	Cross-sectional	Human and dog samples positive for *Ancylostoma spp.*	128 human *Ancylostoma spp.* positive samples, 33 dog *Ancylostoma spp.* positive samples	Multiplex qPCR, qPCR	ITS-1, 2 region	*-* ***A. ceylanicum***: 26·6% [(95% CI 18·8–34·3) (34/128)] - Mixed infections (*A. duodenale* and *N. americanus*): 13.2% [(95% CI 8.7-17.6 (30/228)] - Mixed infections (*N. americanus and A. ceylanicum*): 4.0% [95% CI (1.4-6.5) (9/228)] - Mixed infections (*A. duodenale and A. ceylanicum): 4.8%* [95% CI (2.0-7.6) (11/228)] - Mixed infections (*N. americanus, A. ceylanicum* and *A. duodenale): 4.4%* [95% CI (1.7-7.1) (10/228)]	*- A. ceylanicum:* 36·4% [(95% CI 19·04–3·69) (12/33)]	*18*
Bradbury et al (2017) [62]	Solomon Islands	Study in two villages, all residents invited to participate, egg positive by Kato-Katz method	Cross-sectional	66 of 170 hookworm microscopy samples selected	66 human faecal samples	Multiplex PCR	ITS-1,2 and 5.8s region	*-* ***A. ceylanicum***: 16·7% (11/66) *- N. americanus:* 81·8% (54/66) - Mixed (*A. ceylanicum* and *N. americanus*): 1·5% (1/66)	*Not assessed*	*18*
Ngcamphalala et al (2020) [48]	South Africa	Stool samples of stray dogs collected from 5 centers. Human stool samples collected from 2 primary schools; egg positive by modified Wisconsin sugar flotation method	Cross-sectional	Samples included for sequencing are those with multiple bands after EcoRII restriction, suspected to be non*-A. caninum* species.	3 human faecal samples, 27 dog faecal samples	PCR, PCR-RLFP	ITS1 and 5·8S	**Sequencing** ***- A. caninum***: 100·0% (3/3)	**Sequencing** *- A. caninum:* 81·5% (22/27) - *A. braziliense*: 11·1% (3/27) - Mixed (*A. caninum* & *A. braziliense*): 7·4% (2/27)	15
Jiraanankul et al (2011) [38]	Thailand	Survey of adult and children in a rural community, egg positive by wet preparation, Kato-Katz method and water-ethyl acetate sedimentation technique	Cohort study	50 of 58 microscopy positive samples were successfully amplified	58 human faecal samples	PCR	ITS-1,2 and 5·8s region	*-* ***A. ceylanicum***: 4·0% *(2/50)* *- N. americanus:* 92·0% (46/50) *- A. duodenale:* 2·0% (1/50) - Mixed (*N. americanus* and *A. ceylanicum):* 2·0% *(1/50)*	*Not assessed*	*18*
Areekul et al (2010) [23]	Thailand	Cross-sectional survey in a rural community, stool samples were randomly collected from schoolchildren and dogs, hookworm eggs positive by formalin-ether concentration technique.	Cross-sectional	Samples positive for *Trichuris spp.* by microscopy	56 human faecal samples, 17 dog faecal samples.	PCR	ITS-1, SSU rRNA region	*-* ***T. vulpis***: 10·7% (6/56) *- T. trichiura:* 100% (56/56) - Mixed infections (*T. vulpis* and *T. trichiura)*: 10·7% (6/56)	- ***T. trichiura***: 71·4% (10/14) - *Trichuris spp*.: 82·4% (14/17) - *T. vulpis:* 28·6% (4/14)	*18*
Traub et al (2008) [50]	Thailand	Survey of dogs and humans from temple communities with faecal samples randomly collected, egg positive by zinc sulphate and sodium nitrate flotation method	Cross-sectional	Human- all microscopy positive samples Dogs-122 of 133 microscopy positive samples were successfully amplified	7 human faecal samples, 122 dog faecal samples.	PCR, PCR-RFLP	ITS-1,2 and 5·8s region	*-* ***A. ceylanicum***: 28·6% (2/7) *- N. americanus: 71·4% (5/7)*	*- A. ceylanicum:* 77·0% *(94/122)* *- A. caninum:* 9·0% *(11/122)* - Mixed infections (*A. ceylanicum* and *A. caninum*): 14·0% (17/122)	*18*
Webster et al (2022) [32]	Thailand-Myanmar border	Cross-sectional study as part of an enhanced premigration health program including participants >=6months	Intergrated to a larger program	All samples collected	1835 human faecal samples	qPCR	ITS-1, 2 region	- ***A. ceylanicum***: 5·0% (89/1835) *- A. lumbricoides:* 39·0% (726/ 1835) *- T. trichiura:* 32·0% (598/1835) *- N. americanus:* 26·0% (84/1835) *- G. lamblia:* 22·0% (403/1835) - Mixed infections (more than one organism): 41% (756/1835)	*Not assessed*	*18*
Stracke et al (2019) [36]	Timor-Leste, Cambodia	Validation study for multiplexed quantitative PCR (qPCR), in Timor-Leste school children from six primary schools, In Cambodia participants from ten remote villages	Testing/validation of a diagnostic tool	All samples collected	462 human faecal samples from Timor-Leste, 302 faecal samples from Cambodia.	Multiplexed tandem PCR	ITS-2 region	**Timor-Leste** *-* ***A. ceylanicum***: 1·1% (5/462) - *A*. *lumbricoides:* 33·5% *(155/462)*, - *T*. *trichiura:* 2·4% (*11/462)* *- N*. *americanus:* 10·4% *(48/462)* **Cambodia** - ***A. ceylanicum***: 8·6% (26/302) - *N*. *americanus:* 65·9% (199/302)	*Not assessed*	*18*
Stracke et al (2021) [49]	Thailand	A total of 273 faecal samples from 2- to 6-year-old pre-school and school-aged children, Kato Katz thick smear	Cross-sectional	All samples collected	273 faecal samples	multiplexed-tandem qPCR	ITS-2 region	***A.ceylanicum*:**1·1% (3/273) - *A*. *lumbricoides:* 39·2% *(107/273)*, - *T*. *trichiura:* 36·6% (*100/273)* *-* Mixed infections (*A.lumbricoides* and *T.trichiura*): 24.2% (66/273) *-* Mixed infections (*A. lumbricoides*, *T. trichiura* and *A. ceylanicum*): 0.7% (2/273) *-* Mixed infections (*T. trichiura* and *A. ceylanicum*): 0.4% (1/273)	*Not assessed*	18
Bui et al (2021) [63]	Vietnam	Stool samples collected from residents of a province, egg positive by Kato-Katz	Cross-sectional	Samples positive for hookworm spp. by microscopy	48 human faecal samples	Semi-nested PCR-RLFP	ITS-1,2 and 5·8s region	*-* ***A. ceylanicum:*** 31·3% (15/48) *- N. americanus:* 47·9% (23/48) - Mixed (*A. ceylanicum* & *N. americanus):* 20·8% (10/48)	*Not assessed*	18
Hughes et al (2023) [53]	Vietnam	Cross-sectional study of primary school students in remote regions. Schools randomly selected from a list of eligible schools	Cross-sectional	All samples collected were not subject to qPCR analysis	120 samples per school. 7710 of 8730 (88.3%) stool samples collected were analysed by PCR	qPCR	ITS-1,2	- Overall STH: 14.9% [(95% CI 11.3-18.42) (1149/7710)] - All hookworm: 14.1% [(95% CI 10.6-17.6) (1087/7710)] ***- A. ceylanicum***: 0.6% [(95% CI 0.4-0.8) (46/7710)] *- N. americanus*: 13.7% [(95% CI 10.2-17.2) (1056/7710)] *- A. duodenale*: 0.06% [(95% CI 0.00-0.1) (5/7710)] *- A. lumbricoides*: 0.2% [(95% CI 0.03-0.5) (15/7710)] *- T. trichiura*: 0.7% (95% CI 0.3-1.1) - Mixed infections (two STH species): 0.9% (69/7710)	*Not assessed*	18

* PCR-polymerase chain reaction.

^&^ RFLP-Restriction Fragment Length Polymorphism.

^^^ ITS-Internal Transcribed Spacer.

^¶^ *T. vulpis* was found only in Cameroon.

^§^ Quality score-The critical appraisal tool for cross-sectional studies (AXIS) has a total of 20 points.

^£^ PCR analysis was performed on faecal samples in all studies, with the exception of [56], which examined adult worms.

^±^ COX 1-Cytochrome c oxidase subunit 1.

^#^ qPCR-quantitative PCR.

^||^ SSU rNA-Small subunit ribosomal ribonucleic acid.

Among hospital-based studies, 68.7% (11 of 16) followed a case-study design, while four studies (25%, 4 of 16) utilised preexisting laboratory or patient’s samples [64–67], with one study sampled from both a hospital and a village [68]. These studies included symptomatic patients, with samples comprising of adult worms [64,69–75] and faeces [65,67,68,73,76–78]. Five studies (three from France and two from Japan) reported travel histories to endemic countries such as Thailand [73], Lao PDR [73,76], Myanmar [77], Malaysia [76], India [76], West Indies [79], Pakistan, Cote d‘Ivoire, Colombia, Pakistan, French Guiana [66] and Papua New Guinea [76]; while three mentioned contact with animals [69,71,72] (Table 2).

**Table 2:** Summary of hospital/laboratory-based studies included in the review.

Citation	Country of origin	Clinical history/symptoms	Method of diagnosis	Type of sample analysed by PCR	Number of cases	Species detected	Travel history/animal contact	Quality score*
Koehler et al (2013) [67]	Australia	Stool samples of humans with history of gastrointestinal disorders were tested in 2 laboratories	PCR* based Single-strand conformation polymorphism analysis	Faecal	Two positive cases of 12 tested	*A. ceylanicum*	Recent travel history not recorded	6^†^
Nath et al (2024) [78]	Bangladesh	Recurrent diarrhoea and weakness	PCR	Faecal	One case	*A. ceylanicum*	Recent travel history not recorded	5
Furtado et al (2020) [65]	Brazilian states	–	Conventional PCR	Faecal	One case	*A. caninum*	Recent travel history not recorded	6
Poppert et al (2017) [75]	Colombia	Loss of vision	PCR, sequencing	Adult worm was destroyed during surgical removal. intraoperative rinsing fluid was used	One case	*A. ceylanicum*	Recent travel history not recorded	8
Brunet et al (2015) [77]	France	Fever, vomiting, dyspnoea, bloody diarrhoea and weight loss. Pruritic erythematous macules on buttocks while in Myanmar	PCR	Faecal	One case	*A. ceylanicum*	Returned from Myanmar	7
Gerber et al (2021) [66]	France	Samples of symptomatic patients who have travelled from endemic countries	PCR	Faecal	3/34	*A. ceylanicum*	Returned from Pakistan, Cote d‘Ivoire, Colombia, Pakistan and French Guiana	6
Joncour et al (2012) [79]	France	Itchy rash, persistent pruritis	PCR, DNA sequencing	Larvae from skin scrapings	One case	*A. braziliense*	Returned from the West Indies	7
Romano et al (2021) [71]	Italy	Abdominal pain, vomiting, bloating	PCR	Adult worm	One case	*A. suum*	History of rearing chickens and pigs	7
Dutto M et al (2013) [72]	Italy	Found one worm in his stool	PCR-RFLP^&^	Adult worm	One case	a hybrid genotype-*A. suum/lumbricoides*	No history of travel, pig farmer	7
Arizono et al (2010) [64]	Japan	–	PCR	Adult *Ascaris* worms	Ascaris worms obtained from 9 patients, 3 isolates were of pig origin	*A. suum*	Recent travel history not recorded	6
Yoshikawa et al (2018) [76]	Japan	Three cases had abdominal pain, diarrhoea	PCR	Faecal	4 cases	*A. ceylanicum*	Case 1-returned from Malaysia Case 2-returned from Papua New Guinea Case 3-returned from Lao PDR Case 4-returned from India	7
Nishioka et al (2024) [74]	Japan	Asymptomatic	PCR-RFLP	Worm collected by colonoscopy	One case	*A. suum*	Recent travel history not recorded	7
Jung et al (2020) [69]	South Korea	Moderate eosinophilia	PCR	Worm	One case	*A. caninum*	Patient owns a dog	6
Kaya et al (2016) [73]	Japan	Intermittent diarrhoea, eosinophilia.	PCR	Adult worm	One case	*A. ceylanicum*	Returned from Thailand and Lao PDR	7
Ngui et al (2014) [70]	Malaysia	Upper GI bleed (blood in stool)	PCR, DNA sequencing	Worm	One case	*A. ceylanicum*	Recent travel history not recorded	*5*
Phosuk et al (2013) [68]^$^	Thailand	–	PCR	10 larval hookworm samples from faecal agar plate cultures of patients and 20 from community participants	Three positive cases	*A. ceylanicum*	Recent travel history not recorded	*6*

* Quality score- JBI critical appraisal tool for case studies and cross-sectional studies has a total of 8 points. The JBI critical appraisal tool for cross-sectional studies was used for [64–68].

^$^ Samples were collected from both hospital patients and community participants.

*PCR-polymerase chain reaction.

^&^ RFLP-Restriction Fragment Length Polymorphism.

Polymerase chain reaction (PCR) methods commonly targeting genes such as Internal transcribed spacer-1,2 regions (ITS), mitochondrial cytochrome c oxidase subunit I (COX1), 5·8S, 18S and 28S ribosomal ribonucleic acid (rRNA) were used for species confirmation. In community studies, 45·7% (16 of 35) limited PCR testing to microscopy-positive samples, while 31·4% studies (11 of 35) analysed all samples (Table 1). In some cases (8·6%, 3 of 35), a subset of microscopy-positive samples was randomly selected for PCR [36,44,52]. PCR was conducted in faeces (80·4%, 41 of 51 studies) and in adult worms (15·7%, 8 of 51 studies). The largest surveys were in Myanmar and Thailand, focussing on refugees and employing PCR on all samples [31,32].

### Quality assessment of studies

Majority (91.4%, 32 of 35) of the community-based studies scored 17 points or higher out of a total of 20 points, indicating high quality. Among hospital-based studies, 87.5% (14 of 16) scored 6 points or higher out of a total of 8 points, also reflecting good quality. Details of the quality assessment of the studies are in S2, S3 and S4 Tables.

### Evidence of cross-host species infections

#### Occurrence of zoonotic STH in humans.

Notably, *A. ceylanicum* was the most-reported zoonotic STH species, appearing in 66·7% (34 of 51) of the studies. Its distribution spanning 16 countries, mainly in SEA (61·8%, 21 of 34), with additional occurrences noted in the Solomon Islands, India, Bangladesh, Ecuador, Colombia, France and Australia (Fig 2). Case studies were reported from Japan, France, Malaysia, Bangladesh and Colombia [70,73,75–78] (Table 2).

Twenty studies reported its frequency as a percentage of hookworm positives with proportions ranging from 2·6% [55] to 46·0% [42]. Although *A. ceylanicum* generally appeared to be a minor infection, in four studies its positivity rate was comparable to *N. americanus* [39,40,42,63]. Ten studies, which analysed all collected samples by PCR, found its contribution to the overall prevalence, ranging from 1·1% to 14·8% [31,32,35,36,40,41,55,56,68,80] (Table 1). Other zoonotic STH species observed in SEA included *A. braziliense* (3·4%, 1 of 29 studies), *T. vulpis* (6.9%, 2 of 29 studies), *A. caninum* (3.4%, 1 of 29 studies), and *A. suum* (6.9%, 2 of 29 studies), although their presence was more sporadic.

Reports of zoonotic STH in humans were found to be documented infrequently across other regions globally. In Africa, *A. caninum* [48] and *T. vulpis* [34] in humans were identified in one study each. Likewise, South America witnessed four studies reporting *A. ceylanicum* [39,40,55,75] and one reporting *A. caninum* [65]. In Oceania, *A. ceylanicum* was reported in two studies [62,67]. In South Asia, it was documented in two studies [44,78], and *A. caninum* [33] was reported in another. Finally, in Europe, two studies identified *A. ceylanicum* [66,77], two studies found *A. suum* [71,72], and one study detected *A. braziliense* [79] (Figs 2 and S2).

*A. braziliense* was reported in a tribal population in Laos [37], with another occurrence reported in a case study in France [79]. *A. caninum* was identified in human populations in India [33], Brazil [65], South Africa [48], Lao PDR [58] and in a case study in South Korea [69]. *T. vulpis* was detected in human faeces in two studies in SEA and one in Cameroon [23,34,43]. *A. suum* in humans occurred in one community study in Indonesia [47], in two case studies conducted in Italy, along with another study in a hospital in Japan where samples from patients were identified as *A. suum* [64,71,72].

#### Morbidities linked with zoonotic STH infections.

Clinical case studies documented zoonotic STH infections in humans, presenting with gastrointestinal disturbances (diarrhoea, vomiting, blood in stools, constipation), fever, eosinophilia, difficulty in breathing, and weight loss [66,67,70,71,73,76,77]. One case of *A. braziliense*, confirmed by PCR on two larvae obtained from skin scrapings, presented with itchy rash and persistent pruritic but no gastrointestinal symptoms [79]. In another case of *A. ceylanicum* identified by PCR of larvae, involved pruritic erythematous macules and gastrointestinal symptoms [77]. Two asymptomatic cases were detected through routine testing after returning from abroad [76] (Table 2).

#### Occurrence of human STH species in animals.

Only nine (17·6%) studies [23,37,40,42,43,51,52,57,60] have reported the presence of human STH species in animals. Instances where human STH species were identified in animals involved dogs, cats, and pigs. The occurrence of *T. trichiura* (among *Trichuris spp.* infected dogs and cats) ranged from 26·7% to 71·4% in Malaysia, Thailand, and Myanmar [23,43,60].

*N. americanus* was found in 12.6% (10/79 samples) of dog stools in Ecuador [40], 1 of 18 hookworm samples in Laos [52], and in 47% of dogs (20/43 samples) and 56% of pigs (5/9 samples) in Ghana [16]. *A. duodenale* was found in 6.3% (5/79 samples) of dog stools in Ecuador [40] and 1 of 70 dog hookworm-positive dog samples in Kenya [57]. In India, 31 dog-derived *Ascaris* egg samples matched the digestion pattern of human-derived *Ascaris* by PCR-Restriction Fragment Length Polymorphism, with five showing 100% homology with human *Ascaris* eggs and the adult worm [51] (Figs 3 and S3).

### Human-animal sympatric studies

Among the studies described above, eleven studies systematically explored human and animal populations in shared environments, predominantly investigating dogs and cats with humans [23,33,37,40,42,43,45,48,50–52]. When *T. vulpis* was detected in humans, notably dogs and cats, in the same environment showed high infection rates ranging from 28·6% [23] to 43·2% [43]. Interestingly, in these studies, both dogs and humans were also found to be infected with *T. trichiura* with prevalences ranging 56·8% -71·4% and 98·7-100·0% respectively [23,43]. Similarly, when humans were infected with *A. ceylanicum,* [37,42,45,50,52] correspondingly, dogs showed high infection rates ranging from 38·9% to 90·0%. In India, *A. caninum* was found in humans, with dogs also showing a high infection rate of 76·5% [33]. Furthermore, when dogs were infected with *N. americanus* with prevalences 1·1% - 22·2%, humans also exhibited high infection rates ranging from 47·6% to 82·4% [37,42,52]. Similarly, in areas where dogs were infected with *A. duodenale*, humans were also found to be infected with the same species [40]*.*

## Discussion

This systematic review consolidates evidence of STH cross-host species infections, shedding light on their distribution and diversity. Analysing 51 studies on infections from stool and whole worm samples, the notable presence of zoonotic hookworm infections in humans is highlighted, with *A.ceylanicum*, being a key source of human infections in SEA. Additionally, other zoonotic STH, such as *A.caninum*, *A. braziliense*, *A. suum* and *T.vulpis* were reported sporadically worldwide suggesting an under-recognised global issue. Despite genetic evidence of cross-host infections of *Ascaris* and *Trichuris spp.* between humans and pigs, fewer studies have explored this. The role of animals as reservoirs or carriers for human STH remains under-investigated. Our findings stress the importance of sampling in sympatric environments to better understand these dynamics and underscore the need for representative data, integrating molecular methods and fostering cross-sector collaboration to address animal reservoirs.

Majority of the human studies in this review focussed on zoonotic hookworms, particularly *A. ceylanicum* predominantly reported in SEA, but also in other regions. *A. caninum* and *A. braziliense*, were less commonly reported, although they are known to be widely distributed among dogs in tropical regions [81–88]. With dog ownership averaging 130 dogs per 1000 people globally [89], current data may underestimate the true occurrence of these zoonotic hookworm infections in humans. This raises the possibility that observed human hookworm infections could include contributions from *A. ceylanicum*, *A. caninum* and *A. braziliense*, misidentified as *A. duodenale*. Although zoonotic hookworm infections in humans rarely constitute a major proportion of overall STH or hookworm positives, even low levels of cross-host species infection may have the potential to maintain transmission between humans and animal reservoirs, perpetuating the risk of re-infection and hindering efforts to achieve disease elimination. The emergence of reduced anthelmintic efficacy in humans [90], combined with resistance to benzimidazole [91,92] as seen in *A.caninum* in dogs in the United States of America [92–94], highlights potential future challenges.

The review also sheds light on the zoonotic potential of *T. vulpis* and *A. suum* although in a limited number of studies. *T. vulpis*, primarily found in dogs was detected in human faecal samples in Cameroon [34], Malaysia [43] and Thailand [23]. Similarly, *A. suum*, a pig helminth, was identified in humans in Italy [71,72], Japan [64] and Indonesia [47]. Despite genetic studies demonstrating hybridisation between *A. lumbricoides* and *A. suum* [9,95,96] and between *T. trichiura* and *T. suis*, confirming distinct species with high genetic variation [97,98], suggesting cross-species transmission dynamics between humans and pigs [11,12,97,99], studies exploring these interactions remain scarce. Given the prevalence of small-scale pig farming globally, the role of pigs as potential reservoirs for zoonotic STH warrants further investigation. Additionally, coprophagy in animals can facilitate parasite transmission making them an important part of the transmission cycle.

Only a limited number of studies have investigated the presence of human STH in animals, but this lack of investigation does not imply their absence. Studies found the presence of *T. trichiura* [23,43,60]*, N. americanus* [16,37,40,42,52], *A. duodenale* [40,57] and *A. lumbricoides* in dogs, cats and pigs.

The presence of animal reservoirs could significantly hinder eliminate efforts, which largely rely on mass drug administration (MDA). This necessitates exploring STH transmission through a One Health lens. For example, a modelling study demonstrated that extending MDA to dogs could significantly reduce human *A. ceylanicum* prevalence to less than 1·0% with just 25–50% deworming coverage of dogs by 2030 [100]. Additionally, the studies of humans and animals in shared environments also highlighted coinfection of zoonotic and human STH species in both populations, further emphasising the potential for cross-host transmission in areas of human-animal coexistence.

Understanding the dynamics of cross-host STH infections remains complex. Evidence is needed to confirm whether zoonotic and human STH can complete their life cycles in alternative hosts. Zoonotic STH eggs may mature and reproduce in humans or pass through the human body without causing any harm, and human STH eggs may behave similarly in animals. Although uncertain whether other zoonotic STHs can complete their life cycles in humans, the presence of viable zoonotic hookworm eggs and adult worms seen in the studies of this review suggests potential for onward transmission. Additionally, animals may also serve as mechanical transmitters or transport hosts [15,16] likely contributing to STH transmission

Hospital based studies on symptomatic patients and those with travel history confirmed the clinical relevance of zoonotic STH, particularly *A. ceylanicum*, *A. suum*, *and A. caninum*. Common clinical manifestations include gastrointestinal disturbances, fever, eosinophilia, respiratory difficulties, and weight loss [66,67,69–71,73,76,77]. Zoonotic hookworm infections are also linked to cutaneous larva migrans, though evidence is limited because of underreporting and infrequent investigation of zoonotic hookworms in most settings. Although these studies may not represent the broader distribution of infection, the presence of patent eggs and worms confirmed by molecular analyses highlights the potential of zoonotic STH to cause certain morbidities in humans.

This study has important limitations. Despite, recent community-based studies indicating increasing interest in exploring the occurrence of cross-host species infections, in this review we could not establish its true extent due to heterogeneity in study designs, diverse sampling strategies, limited geographic representativeness, and variations in sample selection criteria for molecular analyses. Sample bias, especially in studies with voluntary participation, hindered drawing comprehensive conclusions on the burden of cross-host species infections in humans and animals.

## Recommendations

The scarcity of information regarding cross-host species infections at present could be attributed to limited exploration, related to morphological examination of eggs, hindering species identification. To address this gap, it is essential to strengthen surveillance by incorporating molecular methods and fostering cross-sector collaboration through a One health approach. Moreover, studies focusing on *A. ceylanicum* are concentrated in SEA, targeting rural communities with high hookworm prevalence and including households with domestic animals. Expanding research to diverse geographical regions beyond SEA and conducting studies in sympatric environments where humans and animals coexist closely are important, as these settings are key interfaces for cross-species transmission. As global initiatives aim to reduce STH morbidity by 2030, improved data on cross-host species infections are essential for informed interventions and improved public health outcomes.

## Supporting information

S1 Checklist PRISMA checklist. (DOCX)

S1 Table Detailed search strategy. (DOCX)

S1 Data Data dictionary of extracted variables. (DOCX)

S1 Fig Number of studies per year. (DOCX)

S2 Data Articles retrieved from each database. (XLSX)

S2 Table Quality assessment AXIS tool. (DOCX)

S2 Fig Zoonotic STH in humans. (DOCX)

S3 Data Full-text reviewed articles. (XLSX)

S3 Table Quality assessment JBI checklist. (DOCX)

S3 Fig Human STH in animals. (DOCX)

S4 Table Quality assessment of case reports JBI checklist. (DOCX)
