# Supplementary material for: The occurrence of cross-host species soil-transmitted helminth infections in humans and domestic/livestock animals: A systematic review
Source: PLOS Glob Public Health. 2025 Aug 12;5(8):e0004614. doi: 10.1371/journal.pgph.0004614 (PMC12342315; doi:10.1371/journal.pgph.0004614)
Supplement: S1 Table — (DOCX) [file pgph.0004614.s002.docx]

**Timelines of Data Extraction**

“The initial database search was carried out by UM between June and August 2023. De-duplication was performed independently by both reviewers (UM and RK) in September 2023, with discrepancies resolved through discussion. Title and abstract screening (October–December 2023) and full-text screening (January–April 2024) were conducted independently and in duplicate. During screening, reviewers were initially blinded to each other’s decisions; discrepancies were resolved through consensus, with RS consulted when needed. Quality and risk of bias assessments were conducted independently in May–June 2024. An updated search was performed by UM in December 2024, followed by independent title and abstract, and full text screening (December 2024-January 2025) .”

# **S1 Table. Detailed search strategy and records retrieved from each database.**

| **Database searched** | **Search strategy** | **Records** |
| --- | --- | --- |
| Medline | ((("Ascaris lumbricoides" or "Trichuris trichiura" or "Ancyclostoma duodenale" or "Necator americanus") and ("small ruminants" or cow or cattle or pig or swine or dogs or canine or cats or feline)) or (("Ascaris suum" or "Ancylostoma ceylanicum" or "Ancylostoma caninum" or "Ancylostoma braziliense" or "Trichuris tulips" or "Trichuris suis") and Humans)).mp. [mp=title, book title, abstract, original title, name of substance word, subject heading word, floating sub-heading word, keyword heading word, organism supplementary concept word, protocol supplementary concept word, rare disease supplementary concept word, unique identifier, synonyms, population supplementary concept word, anatomy supplementary concept word] | 1254 |
| Web of Science | **((TS=("Ascaris lumbricoides" OR "Trichuris trichiura" OR "Ancyclostoma duodenale" OR "Necator americanus")) AND TS=("small ruminants" OR cow OR cattle OR pig OR swine OR dogs OR canine OR cats OR feline)) OR (TS=("Ascaris suum" OR "Ancylostoma ceylanicum" OR "Ancylostoma caninum" OR "Ancylostoma braziliense" OR "Trichuris vulpis" OR "Trichuris suis") AND TS=Humans)** | 1539 |
| PubMed | (((((("Ascaris lumbricoides") OR ("Trichuris trichiura")) OR ("Ancyclostoma duodenale")) OR ("Necator americanus")) AND ((((("small ruminants") OR (cow OR cattle)) OR (pig OR swine)) OR (dogs OR canine)) OR (cats OR feline))) OR (((((("Ascaris suum") OR ("Ancylostoma ceylanicum")) OR ("Ancylostoma caninum")) OR ("Ancylostoma braziliense")) OR ("Trichuris vulpis")) OR ("Trichuris suis"))) AND (Humans) | 1402 |

Two additional papers were not identified through the initial database searches but were included based on their relevance to the review objectives. The first paper by Chin et al [1] was identified through citation tracking, as it was cited in a paper by Mohd-Shaharuddin et al [2], which was captured during the database search. The second paper by Webster et al [3] was linked to another included study by O’Connell et al [4] and was identified during full-text review and cross-referencing.

**References**

1. Chin YT, Lim YAL, Chong CW, Teh CSJ, Yap IKS, Lee SC, et al. Prevalence and risk factors of intestinal parasitism among two indigenous sub-ethnic groups in Peninsular Malaysia. Infect Dis Poverty. 2016;5(1):77.

2. Mohd-Shaharuddin N, Lim YAL, Hassan NA, Nathan S, Ngui R. Molecular characterization of Trichuris species isolated from humans, dogs and cats in rural community Peninsular Malaysia. Acta Trop. 2019;190:269–72.

3. Webster JL, Stauffer WM, Mitchell T, Lee D, O’Connell EM, Weinberg M, et al. Cross-sectional assessment of the association of eosinophilia with intestinal parasitic infection in U.S.-bound refugees in Thailand: prevalent, age dependent, but of limited clinical utility. Am J Trop Med Hyg. 2022;106(5):1552–9.

4. O’Connell EM, Mitchell T, Papaiakovou M, Pilotte N, Lee D, Weinberg M, et al. Ancylostoma ceylanicum hookworm in Myanmar refugees, Thailand, 2012–2015. Emerg Infect Dis. 2018 Aug 1;24(8):1472–81.
