# Supplementary figures and images for: The occurrence of cross-host species soil-transmitted helminth infections in humans and domestic/livestock animals: A systematic review
Source: PLOS Glob Public Health. 2025 Aug 12;5(8):e0004614. doi: 10.1371/journal.pgph.0004614 (PMC12342315; doi:10.1371/journal.pgph.0004614)

**S1 Fig. Number of studies per year and the cumulative total of included studies.**
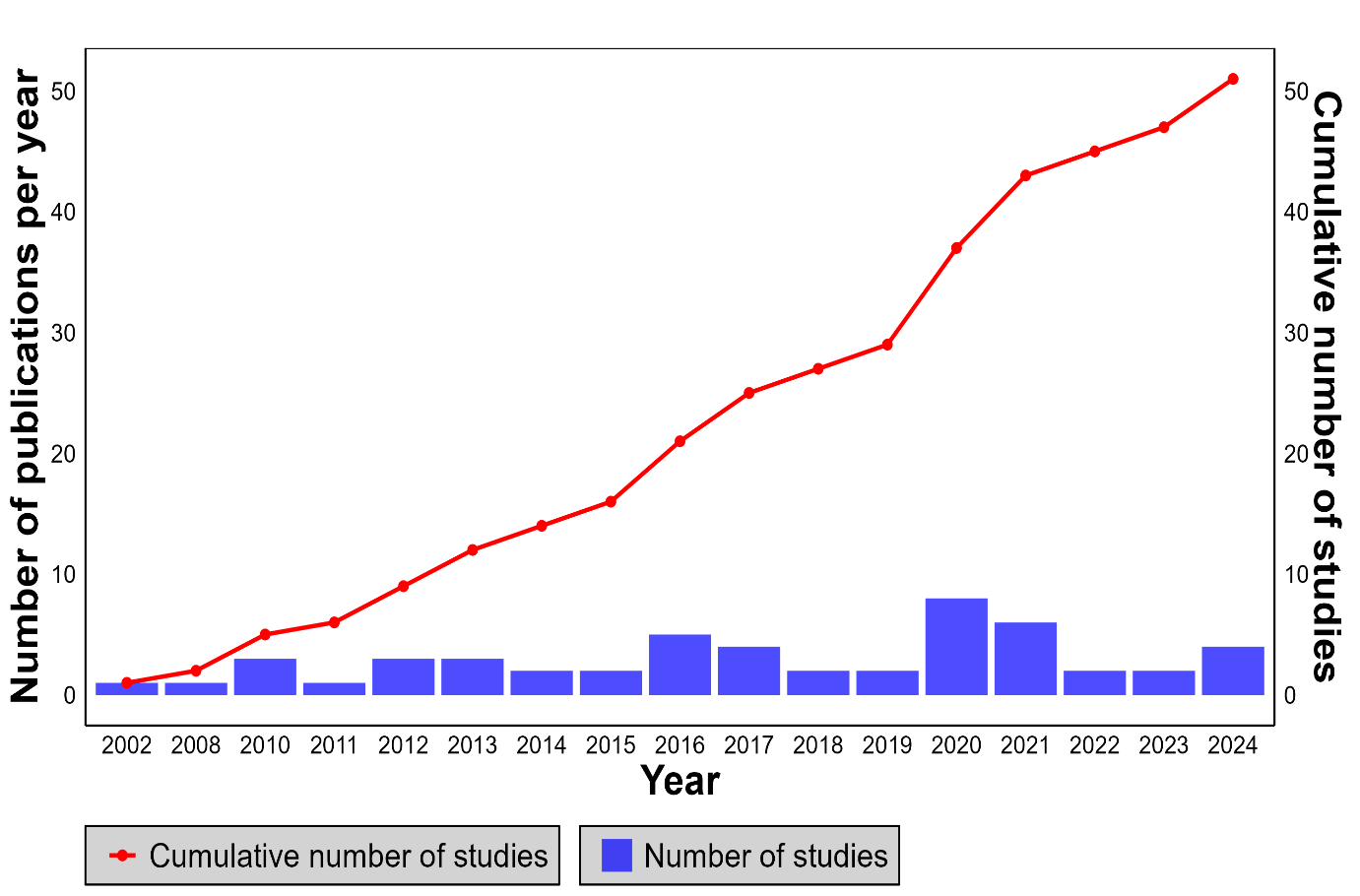

Supplement: S1 Fig — (DOCX) [file pgph.0004614.s004.docx]
