# Supplementary material for: The occurrence of cross-host species soil-transmitted helminth infections in humans and domestic/livestock animals: A systematic review
Source: PLOS Glob Public Health. 2025 Aug 12;5(8):e0004614. doi: 10.1371/journal.pgph.0004614 (PMC12342315; doi:10.1371/journal.pgph.0004614)
Supplement: S2 Table — (DOCX) [file pgph.0004614.s006.docx]

**S2 Table. Quality assessment of studies using the AXIS critical appraisal tool for cross-sectional studies**[1]**.**

| **Author and year of publication** | 1. **Were the aims/objectives of the study clear?** | 1. **Was the study design appropriate for the stated aims?** | 1. **Was the sample size justified?** | 1. **Was the target/reference population clearly define? Is it clear who the research was about?)** | 1. **Was the sample frame taken from an appropriate population base so that it closely represented the target/reference population** | 1. **Was the selection process likely to select subjects/participants that were representative of the target/reference population under investigation?** | 1. **Were measures undertaken to address and categorise non-responders?** | 1. **Were the risk factor and outcome variables measured appropriate to the aims of the study?** | 1. **Were the risk factor and outcome variables measured correctly using instruments /measurements that had been trialled, piloted or published previously?** | 1. **Is it clear what used to determined statistical significance and /or precision estimates?** | 1. **Were the methods (including statistical methods) sufficiently described to enable them to be repeated?** | 1. **Were the basic data adequately described?** | 1. **Does the response rate raise concern about non -response bias?** | **14. If appropriate, was information about non-response described?** | **15. Were the results internally consistent?** | **16. Were the results presented for all analyses described in the methods?** | **17. Were the authors discussions and conclusion described by the results?** | **18. Were the limitations of the study discussed?** | **19. If the paper describe there were not funding sources or conflicts of interest that may affect the authors' interpretation of the results?** | **20. Was ethical approval or consent of participants attained?** | **Score** |
| --- | --- | --- | --- | --- | --- | --- | --- | --- | --- | --- | --- | --- | --- | --- | --- | --- | --- | --- | --- | --- | --- |
| Aguilar-Rodríguez et al (2024)[2] | 1 | 1 | 1 | 1 | 1 | 1 | 0 | 1 | 1 | 0 | 1 | 1 | 1 | 0 | 1 | 1 | 1 | 1 | 1 | 1 | 17 |
| Agustina et al (2023)[3] | 1 | 1 | 0 | 1 | 1 | 0 | 0 | 1 | 1 | 0 | 1 | 1 | 1 | 0 | 1 | 1 | 1 | 0 | 1 | 1 | 15 |
| Areekul et al (2010)[4] | 1 | 1 | 1 | 1 | 1 | 1 | 0 | 1 | 1 | 1 | 1 | 1 | 1 | 0 | 1 | 1 | 1 | 1 | 1 | 1 | 18 |
| Ash et al (2017)[5] | 1 | 1 | 1 | 1 | 1 | 1 | 0 | 1 | 1 | 1 | 1 | 1 | 1 | 0 | 1 | 1 | 1 | 1 | 1 | 1 | 18 |
| Aula et al (2020)[6] | 1 | 1 | 1 | 1 | 1 | 1 | 0 | 1 | 1 | 1 | 1 | 1 | 1 | 0 | 1 | 1 | 1 | 1 | 1 | 1 | 18 |
| Aung et al (2017)[7] | 1 | 1 | 1 | 1 | 1 | 1 | 0 | 1 | 1 | 1 | 1 | 1 | 1 | 0 | 1 | 1 | 1 | 1 | 1 | 1 | 18 |
| Boyko et al (2020)[8] | 1 | 1 | 1 | 1 | 1 | 1 | 0 | 1 | 1 | 1 | 1 | 1 | 1 | 0 | 1 | 1 | 1 | 1 | 1 | 1 | 18 |
| Bradbury et al (2017)[9] | 1 | 1 | 1 | 1 | 1 | 1 | 0 | 1 | 1 | 1 | 1 | 1 | 1 | 0 | 1 | 1 | 1 | 1 | 1 | 1 | 18 |
| Bui et al (2021)[10] | 1 | 1 | 1 | 1 | 1 | 1 | 0 | 1 | 1 | 1 | 1 | 1 | 1 | 0 | 1 | 1 | 1 | 1 | 1 | 1 | 18 |
| Calvopina et al (2024)[11] | 1 | 1 | 0 | 1 | 1 | 0 | 0 | 1 | 1 | 0 | 1 | 1 | 1 | 0 | 1 | 1 | 1 | 1 | 1 | 1 | 15 |
| Chang et al (2020)[12] | 1 | 1 | 1 | 1 | 1 | 1 | 0 | 1 | 1 | 1 | 1 | 1 | 1 | 0 | 1 | 1 | 1 | 1 | 1 | 1 | 18 |
| Chin et al (2016)[13] | 1 | 1 | 1 | 1 | 1 | 1 | 0 | 1 | 1 | 1 | 1 | 1 | 1 | 0 | 1 | 1 | 1 | 1 | 1 | 1 | 18 |
| Conlan et al (2012)[14] | 1 | 1 | 1 | 1 | 1 | 1 | 0 | 1 | 1 | 1 | 1 | 1 | 1 | 0 | 1 | 1 | 1 | 1 | 1 | 1 | 18 |
| (Colella et al (2021)[15] | 1 | 1 | 1 | 1 | 1 | 1 | 0 | 1 | 1 | 1 | 1 | 1 | 1 | 0 | 1 | 1 | 1 | 1 | 1 | 1 | 18 |
| Dunn et al (2020)[16] | 1 | 1 | 1 | 1 | 1 | 1 | 0 | 1 | 1 | 1 | 1 | 1 | 1 | 0 | 1 | 1 | 1 | 1 | 1 | 1 | 18 |
| George et al (2015)[17] | 1 | 1 | 1 | 1 | 1 | 1 | 0 | 1 | 1 | 1 | 1 | 1 | 1 | 0 | 1 | 1 | 1 | 1 | 1 | 1 | 18 |
| George, Geldhof, et al (2016)[18] | 1 | 1 | 1 | 1 | 1 | 1 | 0 | 1 | 1 | 1 | 1 | 1 | 1 | 0 | 1 | 1 | 1 | 1 | 1 | 1 | 18 |
| George, Levecke, et al (2016)[19] | 1 | 1 | 1 | 1 | 1 | 1 | 0 | 1 | 1 | 1 | 1 | 1 | 1 | 0 | 1 | 1 | 1 | 1 | 1 | 1 | 18 |
| Htun et AL (2021)[20] | 1 | 1 | 1 | 1 | 1 | 1 | 0 | 1 | 1 | 1 | 1 | 1 | 1 | 0 | 1 | 1 | 1 | 1 | 1 | 1 | 18 |
| Hughes et al (2023)[21] | 1 | 1 | 1 | 1 | 1 | 1 | 0 | 1 | 1 | 1 | 1 | 1 | 1 | 0 | 1 | 1 | 1 | 1 | 1 | 1 | 18 |
| Inpankaew et al (2014)[22] | 1 | 1 | 1 | 1 | 1 | 1 | 0 | 1 | 1 | 1 | 1 | 1 | 1 | 0 | 1 | 1 | 1 | 1 | 1 | 1 | 18 |
| Jiraanankul et al (2011)[23] | 1 | 1 | 1 | 1 | 1 | 1 | 0 | 1 | 1 | 1 | 1 | 1 | 1 | 0 | 1 | 1 | 1 | 1 | 1 | 1 | 18 |
| Mohd-Shaharuddin et al (2019)[24] | 1 | 1 | 1 | 1 | 1 | 1 | 0 | 1 | 1 | 1 | 1 | 1 | 1 | 0 | 1 | 1 | 1 | 1 | 1 | 1 | 18 |
| Mulinge et al (2020)  [25] | 1 | 1 | 1 | 1 | 1 | 1 | 0 | 1 | 1 | 1 | 1 | 1 | 1 | 0 | 1 | 1 | 1 | 1 | 1 | 1 | 18 |
| Niamnuy et al (2016)[26] | 1 | 1 | 1 | 1 | 1 | 1 | 0 | 1 | 1 | 1 | 1 | 1 | 1 | 0 | 1 | 1 | 1 | 1 | 1 | 1 | 18 |
| Ngcamphalala et al (2020)[27] | 1 | 1 | 0 | 1 | 1 | 0 | 0 | 1 | 1 | 0 | 1 | 1 | 1 | 0 | 1 | 1 | 1 | 1 | 1 | 1 | 15 |
| Ngui et al (2012)[28] | 1 | 1 | 1 | 1 | 1 | 0 | 0 | 1 | 1 | 1 | 1 | 1 | 1 | 0 | 1 | 1 | 1 | 1 | 1 | 1 | 17 |
| O’Connell et al (2018)[29] | 1 | 1 | 1 | 1 | 1 | 1 | 0 | 1 | 1 | 1 | 1 | 1 | 1 | 0 | 1 | 1 | 1 | 1 | 1 | 1 | 18 |
| Traub et al (2008)[30] | 1 | 1 | 1 | 1 | 1 | 1 | 0 | 1 | 1 | 1 | 1 | 1 | 1 | 0 | 1 | 1 | 1 | 1 | 1 | 1 | 18 |
| Traub et al (2002)[31] | 1 | 1 | 1 | 1 | 1 | 1 | 0 | 1 | 1 | 1 | 1 | 1 | 1 | 0 | 1 | 1 | 1 | 1 | 1 | 1 | 18 |
| Sato et al (2010)[32] | 1 | 1 | 1 | 1 | 1 | 1 | 0 | 1 | 1 | 1 | 1 | 1 | 1 | 0 | 1 | 1 | 1 | 1 | 1 | 1 | 18 |
| Sears et al (2022)[33] | 1 | 1 | 1 | 1 | 1 | 1 | 0 | 1 | 1 | 1 | 1 | 1 | 1 | 0 | 1 | 1 | 1 | 1 | 1 | 1 | 18 |
| Stracke et al (2019)[34] | 1 | 1 | 1 | 1 | 1 | 1 | 0 | 1 | 1 | 1 | 1 | 1 | 1 | 0 | 1 | 1 | 1 | 1 | 1 | 1 | 18 |
| Stracke et al (2021)[35] | 1 | 1 | 1 | 1 | 1 | 1 | 0 | 1 | 1 | 1 | 1 | 1 | 1 | 0 | 1 | 1 | 1 | 1 | 1 | 1 | 18 |
| Webster et al (2022)[36] | 1 | 1 | 1 | 1 | 1 | 1 | 0 | 1 | 1 | 1 | 1 | 1 | 1 | 0 | 1 | 1 | 1 | 1 | 1 | 1 | 18 |

Yes=1, No=0

# References

1. Downes MJ, Brennan ML, Williams HC, Dean RS. Development of a critical appraisal tool to assess the quality of cross-sectional studies (AXIS). BMJ Open. 2016;6(12):e011458.

2. Aguilar-Rodríguez D, Seco-Hidalgo V, Lopez A, Romero-Sandoval N, Calvopiña M, Guevara A, et al. Geographic distribution of human infections with zoonotic Ancylostoma ceylanicum and anthropophilic hookworms in Ecuador: a retrospective analysis of archived stool samples. Am J Trop Med Hyg. 2024;110(3):460–9.

3. Agustina KK, Wirawan IMA, Sudarmaja IM, Subrata IM, Dharmawan NS. Ascaris suum – A zoonosis in Bali, Indonesia. Tropical Parasitology. 2023 Dec;13(2):100.

4. Areekul P, Putaporntip C, Pattanawong U, Sitthicharoenchai P, Jongwutiwes S. Trichuris vulpis and T. trichiura infections among schoolchildren of a rural community in northwestern Thailand: the possible role of dogs in disease transmission. Asian Biomed. 2010;4(1):49–60.

5. Ash A, Okello A, Khamlome B, Inthavong P, Allen J, Thompson RCA. Controlling Taenia solium and soil transmitted helminths in a northern Lao PDR village: Impact of a triple dose albendazole regime. Acta Trop. 2017 Oct;174:171–8.

6. Aula OP, Mcmanus DP, Weerakoon KG, Olveda R, Ross AG, Rogers MJ, et al. Molecular identification of Ancylostoma ceylanicum in the Philippines. Parasitology. 2020;147(14):1718–22.

7. Aung WPP, Htoon TT, Tin HH, Sanpool O, Jongthawin J, Sadaow L, et al. First molecular identifications of Necator americanus and Ancylostoma ceylanicum infecting rural communities in Lower Myanmar. Am J Trop Med Hyg. 2017;96(1):214–6.

8. Boyko RH, Marie Harrison L, Humphries D, Galvani AP, Townsend JP, Otchere J, et al. Dogs and pigs are transport hosts of Necator americanus: molecular evidence for a zoonotic mechanism of human hookworm transmission in Ghana. Zoonoses Public Health. 2020;67(5):474–83.

9. Bradbury RS, Hii SF, Harrington H, Speare R, Traub R. Ancylostoma ceylanicum Hookworm in the Solomon Islands. Emerg Infect Dis. 2017;23(2):252.

10. Bui KL, Nguyen TH, Duong HD, Nguyen VL, Nguyen TN, Le LA, et al. Ancylostoma ceylanicum infections in humans in Vietnam. Parasitol Int. 2021;84:102405.

11. Calvopina M, Aguilar-Rodríguez D, DeGroot A, Cevallos W, Lee GO, Lopez A, et al. Anthroponotic and zoonotic hookworm DNA in an Indigenous community in coastal Ecuador: potential cross-transmission between dogs and humans. Pathogens. 2024;13(8):609.

12. Chang T, Jung BK, Sohn WM, Hong S, Shin H, Ryoo S, et al. Morphological and molecular diagnosis of Necator americanus and Ancylostoma ceylanicum recovered from villagers in Northern Cambodia. Korean J Parasitol. 2020;58(6):619.

13. Chin YT, Lim YAL, Chong CW, Teh CSJ, Yap IKS, Lee SC, et al. Prevalence and risk factors of intestinal parasitism among two indigenous sub-ethnic groups in Peninsular Malaysia. Infect Dis Poverty. 2016;5(1):77.

14. Conlan JV, Khamlome B, Vongxay K, Elliot A, Pallant L, Sripa B, et al. Soil-transmitted helminthiasis in Laos: a community-wide cross-sectional study of humans and dogs in a mass drug administration environment. Am J Trop Med Hyg. 2012;86(4):624.

15. Colella V, Khieu V, Worsley A, Senevirathna D, Muth S, Huy R, et al. Risk profiling and efficacy of albendazole against the hookworms Necator americanus and Ancylostoma ceylanicum in Cambodia to support control programs in Southeast Asia and the Western Pacific. Lancet Reg Health West Pac. 2021;16:100258.

16. Dunn JC, Papaiakovou M, Han KT, Chooneea D, Bettis AA, Wyine NY, et al. The increased sensitivity of qPCR in comparison to Kato-Katz is required for the accurate assessment of the prevalence of soil-transmitted helminth infection in settings that have received multiple rounds of mass drug administration. Parasites Vectors. 2020;13(1):1–11.

17. George S, Kaliappan SP, Kattula D, Roy S, Geldhof P, Kang G, et al. Identification of Ancylostoma ceylanicum in children from a tribal community in Tamil Nadu, India using a semi-nested PCR-RFLP tool. Trans R Soc Trop Med Hyg. 2015;109(4):283–5.

18. George S, Geldhof P, Albonico M, Ame SM, Bethony JM, Engels D, et al. The molecular speciation of soil-transmitted helminth eggs collected from school children across six endemic countries. Trans R Soc Trop Med Hyg. 2016;110(11):657–63.

19. George S, Levecke B, Kattula D, Velusamy V, Roy S, Geldhof P, et al. Molecular Identification of Hookworm Isolates in Humans, Dogs and Soil in a Tribal Area in Tamil Nadu, India. PLoS Negl Trop Dis. 2016;10(8):e0004891.

20. Htun LL, Rein ST, Win SY, Soe NC, Thein SS, Khaing Y, et al. Occurrence of gastrointestinal helminths and the first molecular detection of Ancylostoma ceylanicum, Trichuris trichiura, and Trichuris vulpis in dogs in Myanmar. Parasitol Res. 2021;120(10):3619–24.

21. Hughes A, Ng-Nguyen D, Clarke NE, Dyer CEF, Hii SF, Clements ACA, et al. Epidemiology of soil-transmitted helminths using quantitative PCR and risk factors for hookworm and Necator americanus infection in school children in Dak Lak province, Vietnam. Parasites Vectors. 2023;16(1):213.

22. Inpankaew T, Schär F, Dalsgaard A, Khieu V, Chimnoi W, Chhoun C, et al. High prevalence of Ancylostoma ceylanicum hookworm infections in humans, Cambodia, 2012. Emerg Infect Dis. 2014;20(6):976.

23. Jiraanankul V, Aphijirawat W, Mungthin M, Khositnithikul R, Rangsin R, Traub RJ, et al. Incidence and risk factors of hookworm infection in a rural community of central Thailand. Am J Trop Med Hyg. 2011;84(4):594.

24. Mohd-Shaharuddin N, Lim YAL, Hassan NA, Nathan S, Ngui R. Molecular characterization of Trichuris species isolated from humans, dogs and cats in rural community Peninsular Malaysia. Acta Trop. 2019;190:269–72.

25. Mulinge E, Njenga SM, Odongo D, Magambo J, Zeyhle E, Mbae C, et al. Molecular identification of zoonotic hookworms in dogs from four counties of Kenya. J Helminthol. 2020;94:e43.

26. Niamnuy N, Kaewthamasorn M, Congpuong K, Phaytanavanh B, Lohsoonthorn V. Prevalence and associated risk factors of intestinal parasites in humans and domestic animals across borders of Thailand and Lao PDR: focus on hookworm and threadworm. Southeast Asian J Trop Med Public Health. 2016;47(5):901–11.

27. Ngcamphalala PI, Lamb J, Mukaratirwa S. Molecular identification of hookworm isolates from stray dogs, humans and selected wildlife from South Africa. J Helminthol. 2020;94.

28. Ngui R, Lim YAL, Traub R, Mahmud R, Mistam MS. Epidemiological and genetic data supporting the transmission of Ancylostoma ceylanicum among human and domestic animals. PLoS Negl Trop Dis. 2012;6(2).

29. O’Connell EM, Mitchell T, Papaiakovou M, Pilotte N, Lee D, Weinberg M, et al. Ancylostoma ceylanicum hookworm in Myanmar refugees, Thailand, 2012–2015. Emerg Infect Dis. 2018 Aug 1;24(8):1472–81.

30. Traub RJ, Inpankaew T, Sutthikornchai C, Sukthana Y, Thompson RCA. PCR-based coprodiagnostic tools reveal dogs as reservoirs of zoonotic ancylostomiasis caused by Ancylostoma ceylanicum in temple communities in Bangkok. Vet Parasitol. 2008;155(1–2):67–73.

31. Traub RJ, Robertson ID, Irwin P, Mencke N, Thompson RCA. The role of dogs in transmission of gastrointestinal parasites in a remote tea-growing community in northeastern India. Am J Trop Med Hyg. 2002;67(5):539–45.

32. Sato M, Sanguankiat S, Yoonuan T, Pongvongsa T, Keomoungkhoun M, Phimmayoi I, et al. Copro-molecular identification of infections with hookworm eggs in rural Lao PDR. Trans R Soc Trop Med Hyg. 2010;104(9):617–22.

33. Sears WJ, Cardenas J, Kubofcik J, Nutman TB, Cooper PJ. Zoonotic Ancylostoma ceylanicum hookworm infections, Ecuador. Emerg Infect Dis. 2022;28(9):1867–9.

34. Stracke K, Clarke N, Awburn CV, Vaz Nery S, Khieu V, Traub RJ, et al. Development and validation of a multiplexed-tandem qPCR tool for diagnostics of human soil-transmitted helminth infections. PLoS Negl Trop Dis. 2019 Jun 17;13(6):e0007363.

35. Stracke K, Adisakwattana P, Phuanukoonnon S, Yoonuan T, Poodeepiyasawat A, Dekumyoy P, et al. Field evaluation of the gut microbiome composition of pre-school and school-aged children in Tha Song Yang, Thailand, following oral MDA for STH infections. PLoS Negl Trop Dis. 2021;15(7):e0009597.

36. Webster JL, Stauffer WM, Mitchell T, Lee D, O’Connell EM, Weinberg M, et al. Cross-sectional assessment of the association of eosinophilia with intestinal parasitic infection in U.S.-bound refugees in Thailand: prevalent, age dependent, but of limited clinical utility. Am J Trop Med Hyg. 2022;106(5):1552–9.
