# Supplementary material for: The occurrence of cross-host species soil-transmitted helminth infections in humans and domestic/livestock animals: A systematic review
Source: PLOS Glob Public Health. 2025 Aug 12;5(8):e0004614. doi: 10.1371/journal.pgph.0004614 (PMC12342315; doi:10.1371/journal.pgph.0004614)
Supplement: S3 Table — (DOCX) [file pgph.0004614.s009.docx]

**S3 Table. Quality assessment of studies using the JBI checklist for cross-sectional studies (hospital-based studies)**[1]**.**

| **Author and year of publication** | 1. **Were the criteria for inclusion in the sample clearly defined?** | 1. **Were the study subjects and the setting described in detail?** | 1. **Was the exposure measured in a valid and reliable way?** | 1. **Were objective, standard criteria used for measurement of the condition?** | 1. **Were confounding factors identified?** | 1. **Were strategies to deal with confounding factors stated?** | 1. **Were the outcomes measured in a valid and reliable way?** | 1. **Was appropriate statistical analysis used?** | **Score** |
| --- | --- | --- | --- | --- | --- | --- | --- | --- | --- |
| Arizono et al (2010)[2] | 1 | 1 | 1 | 1 | 0 | 0 | 1 | 1 | 6 |
| Furtado et al (2020)[3] | 1 | 1 | 1 | 1 | 0 | 0 | 1 | 1 | 6 |
| Gerber et al (2021)[4] |  |  |  |  |  |  |  |  |  |
| Koehler et al (2013)[5] | 1 | 1 | 1 | 1 | 0 | 0 | 1 | 1 | 6 |
| Phosuk et al (2013)[6] | 1 | 1 | 1 | 1 | 0 | 0 | 1 | 1 | 6 |

Yes=1, No=0

# **References**

1. Moola S, Munn Z, Tufanaru C, Aromataris E, Sears K, Sfetcu R. Chapter 7: Systematic reviews of etiology and risk. JBI Manual for Evidence Synthesis. 2020.

2. Arizono N, Yoshimura Y, Tohzaka N, Yamada M, Tegoshi T, Onishi K, et al. Ascariasis in Japan: is pig-derived Ascaris infecting humans? Jpn J Infect Dis. 2010 Nov;63(6):447–8.

3. Furtado LFV, Dias LT de O, Rodrigues T de O, Silva VJ da, Oliveira VNGM de, Rabelo ÉML. Egg genotyping reveals the possibility of patent Ancylostoma caninum infection in human intestine. Sci Rep. 2020;10(1):1–7.

4. Gerber V, Le Govic Y, Ramade C, Chemla C, Hamane S, Desoubeaux G, et al. Ancylostoma ceylanicum as the second most frequent hookworm species isolated in France in travellers returning from tropical areas. J Travel Med. 2021;28(6):taab014.

5. Koehler AV, Bradbury RS, Stevens MA, Haydon SR, Jex AR, Gasser RB. Genetic characterization of selected parasites from people with histories of gastrointestinal disorders using a mutation scanning-coupled approach. Electrophoresis. 2013;34(12):1720–8.

6. Phosuk I, Intapan PM, Thanchomnang T, Sanpool O, Janwan P, Laummaunwai P, et al. Molecular Detection of Ancylostoma duodenale, Ancylostoma ceylanicum, and Necator americanus in Humans in Northeastern and Southern Thailand. Korean J Parasitol. 2013;51(6):747.
