# Supplementary material for: The occurrence of cross-host species soil-transmitted helminth infections in humans and domestic/livestock animals: A systematic review
Source: PLOS Glob Public Health. 2025 Aug 12;5(8):e0004614. doi: 10.1371/journal.pgph.0004614 (PMC12342315; doi:10.1371/journal.pgph.0004614)

**S3 Fig: Number of studies by country reporting human STH in animals.** The country borders shapefile used as the base layer is the wb_countries_admin0_10m dataset, available from <https://datacatalog.worldbank.org/search/dataset/0038272>, and is licensed under the Creative Commons Attribution 4.0 International (CC BY 4.0) license.


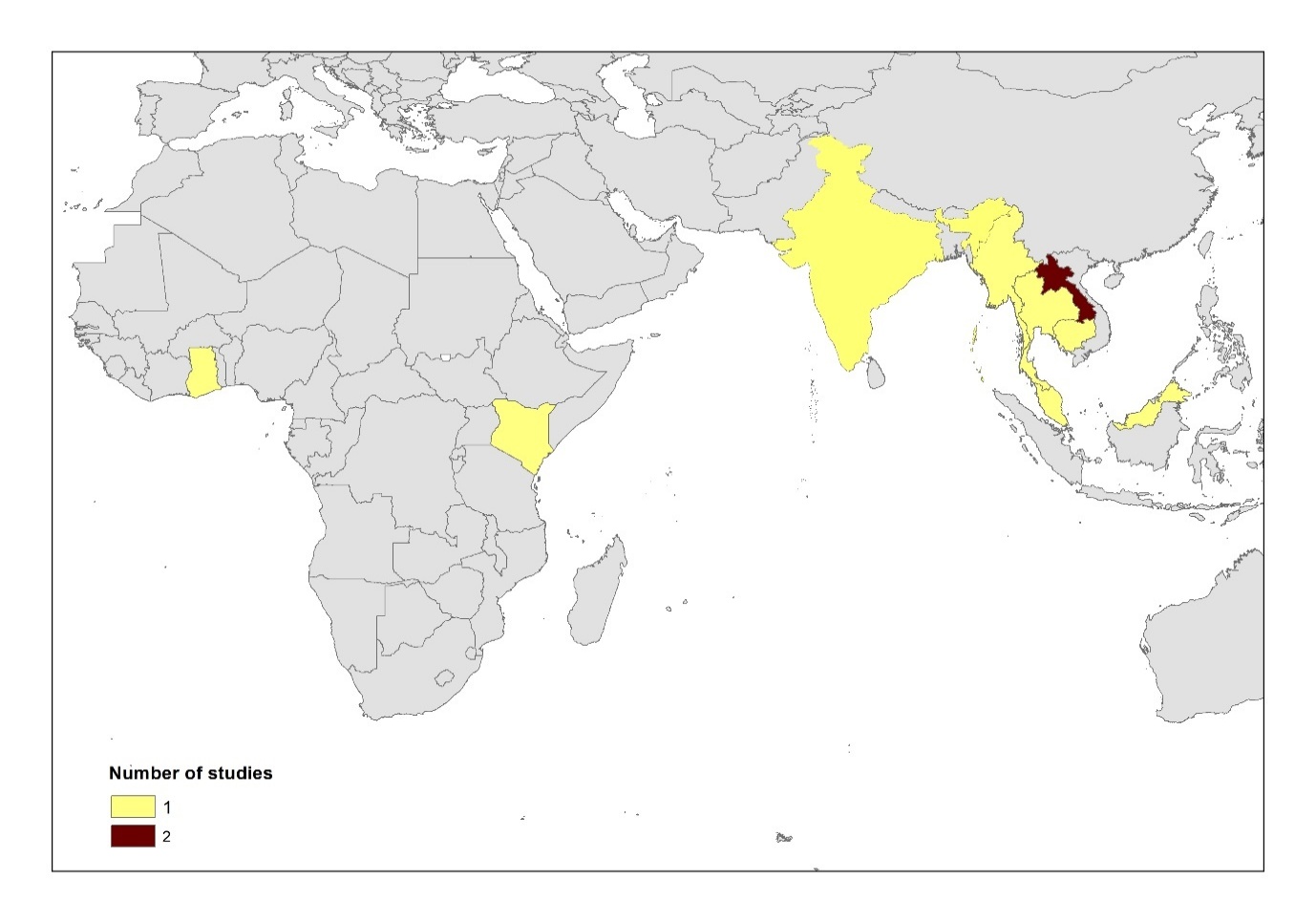

Supplement: S3 Fig — (DOCX) [file pgph.0004614.s010.docx]
