# Supplementary material for: The occurrence of cross-host species soil-transmitted helminth infections in humans and domestic/livestock animals: A systematic review
Source: PLOS Glob Public Health. 2025 Aug 12;5(8):e0004614. doi: 10.1371/journal.pgph.0004614 (PMC12342315; doi:10.1371/journal.pgph.0004614)
Supplement: S4 Table — (DOCX) [file pgph.0004614.s011.docx]

# **S4 Table. Quality assessment of case reports using the JBI checklist for case reports**[1]**.**

| **Author and year of publication** | 1. **Were patient’s demographic characteristics clearly described?** | 1. **Was the patient’s history clearly described and presented as a timeline?** | 1. **Was the current clinical condition of the patient on presentation clearly described?** | 1. **Were diagnostic tests or assessment methods and the results clearly described?** | 1. **Was the intervention(s) or treatment procedure(s) clearly described?** | 1. **Was the post-intervention clinical condition clearly described?** | 1. **Were adverse events (harms) or unanticipated events identified and described?** | 1. **Does the case report provide takeaway lessons?** | **Score** |
| --- | --- | --- | --- | --- | --- | --- | --- | --- | --- |
| Brunet et al (2015)[2] | 1 | 1 | 1 | 1 | 1 | 1 | 0 | 1 | 7 |
| Dutto & Petrosillo (2013)[3] | 1 | 1 | 1 | 1 | 1 | 1 | 0 | 1 | 7 |
| Nath et al (2024)[4] | 1 | 1 | 1 | 1 | 0 | 0 | 0 | 1 | 5 |
| Nishioka et al (2024)[5] | 1 | 1 | 1 | 1 | 1 | 1 | 0 | 1 | 7 |
| Poppert et al (2017)[6] | 1 | 1 | 1 | 1 | 1 | 1 | 1 | 1 | 8 |
| Le Joncour et al (2012)[7] | 1 | 1 | 1 | 1 | 1 | 1 | 0 | 1 | 7 |
| Romano et al (2021)[8] | 1 | 1 | 1 | 1 | 1 | 1 | 0 | 1 | 7 |
| Jung et al (2020)[9] | 1 | 1 | 1 | 1 | 1 | 0 | 0 | 1 | 6 |
| Kaya et al (2016)[10] | 1 | 1 | 1 | 1 | 1 | 1 | 0 | 1 | 7 |
| Ngui et al (2014)[11] | 1 | 1 | 1 | 1 | 0 | 0 | 0 | 1 | 5 |
| Yoshikawa et al (2018)[12] | 1 | 1 | 1 | 1 | 1 | 1 | 0 | 1 | 7 |

Yes=1, No=0

# **References**

1. Moola S, Munn Z, Tufanaru C, Aromataris E, Sears K, Sfetcu R. Chapter 7: Systematic reviews of etiology and risk. JBI Manual for Evidence Synthesis. 2020.

2. Brunet J, Lemoine JP, Lefebvre N, Denis J, Pfaff AW, Abou-Bacar A, et al. Bloody diarrhea associated with hookworm infection in traveler returning to France from Myanmar. Emerg Infect Dis. 2015;21(10):1878.

3. Dutto M, Petrosillo N. Hybrid ascaris suum/lumbricoides (ascarididae) infestation in a pig farmer: a rare case of zoonotic ascariasis. Cent Eur J Public Health. 2013;21(4):224–6.

4. Nath TC, Tusher PC, Siddiki T, Nyema J, Bhattacharjee T, Dey N, et al. Rare case of human *Ancylostoma ceylanicum* infection in Bangladesh. IJID Regions. 2024;11:100376.

5. Nishioka M, Hamabe K, Kunimune Y, Kodama M, Nakahara Y, Okayama N, et al. A case of asymptomatic infection of *Ascaris suum* identified by PCR-restriction fragment length polymorphism and DNA sequence analysis. Diagn Microbiol Infect Dis. 2024;110(1):116444.

6. Poppert S, Heideking M, Agostini H, Fritzenwanker M, Wuppenhorst N, Muntau B, et al. Diffuse Unilateral Subacute Neuroretinitis Caused by Ancylostoma Hookworm. Emerg Infect Dis. 2017;23(2):343–4.

7. Le Joncour A, Lacour SA, Lecso G, Regnier S, Guillot J, Caumes E. Case Report: Molecular Characterization of Ancylostoma braziliense Larvae in a Patient with Hookworm-Related Cutaneous Larva Migrans. Am J Trop Med Hyg. 2012;86(5):843.

8. Romano G, Pepe P, Cavallero S, Cociancic P, Di Libero L, Grande G, et al. Ascariasis in a 75-year-old man with small bowel volvulus: a case report. BMC Infect Dis. 2021;21:1045.

9. Jung BK, Lee JY, Chang T, Song H, Chai JY. Rare Case of Enteric Ancylostoma caninum Hookworm Infection, South Korea. Emerg Infect Dis. 2020;26(1):181.

10. Kaya K, Yoshikawa H, Natakani N, Tomo-Oka F, Fujimoto Y, K I, et al. Ancylostoma ceylanicum hookworm infection in Japanese traveler who presented chronic diarrhea after return from Lao People’s Democratic Republic. Parasitol Int. 2016;65(6 Pt A):737–40.

11. Ngui R, Lim YAL, Ismail WHW, Lim KN, Mahmud R. Case Report: Zoonotic Ancylostoma ceylanicum Infection Detected by Endoscopy. Am J Trop Med Hyg. 2014;91(1):86.

12. Yoshikawa M, Ouji Y, Hirai N, Nakamura-Uchiyama F, Yamada M, Arizono N, et al. Ancylostoma ceylanicum, novel etiological agent for traveler’s diarrhea—report of four Japanese patients who returned from Southeast Asia and Papua New Guinea. Trop Med Health. 2018;46(1).
